# Characterization of NRPS and PKS genes involved in the biosynthesis of SMs in *Alternaria dauci* including the phytotoxic polyketide aldaulactone

**DOI:** 10.1038/s41598-022-11896-0

**Published:** 2022-05-17

**Authors:** Julia Courtial, Jean-Jacques Helesbeux, Hugo Oudart, Sophie Aligon, Muriel Bahut, Bruno Hamon, Guillaume N’Guyen, Sandrine Pigné, Ahmed G. Hussain, Claire Pascouau, Nelly Bataillé-Simoneau, Jérôme Collemare, Romain Berruyer, Pascal Poupard

**Affiliations:** 1 Univ Angers, Institut Agro, INRAE, IRHS, SFR 4207 QuaSaV, 49000 Angers, France; 2 Substances d’Origine Naturelle et Analogues Structuraux, SFR4207 QUASAV, Université d’Angers, Angers, France; 3 ANAN, SFR 4207 QUASAV, Beaucouzé, France; 4 Westerdijk Fungal Biodiversity Institute, Uppsalalaan 8, 3584CT Utrecht, The Netherlands; 5 Laboratory of Virology, Wageningen University and Research, Droevendaalsesteeg 1, 6708 PB Wageningen, The Netherlands

**Keywords:** Secondary metabolism, Microbiology, Plant sciences, Genetics, Comparative genomics

## Abstract

*Alternaria dauci* is a *Dothideomycete* fungus, causal agent of carrot leaf blight. As a member of the *Alternaria* genus, known to produce a lot of secondary metabolite toxins, *A. dauci* is also supposed to synthetize host specific and non-host specific toxins playing a crucial role in pathogenicity. This study provides the first reviewing of secondary metabolism genetic basis in the *Alternaria* genus by prediction of 55 different putative core genes. Interestingly, aldaulactone, a phytotoxic benzenediol lactone from *A. dauci*, was demonstrated as important in pathogenicity and in carrot partial resistance to this fungus*.* As nothing is known about aldaulactone biosynthesis, bioinformatic analyses on a publicly available *A. dauci* genome data set that were reassembled, thanks to a transcriptome data set described here, allowed to identify 19 putative secondary metabolism clusters. We exploited phylogeny to pinpoint cluster 8 as a candidate in aldaulactone biosynthesis. This cluster contains *AdPKS7* and *AdPKS8*, homologs with genes encoding a reducing and a non-reducing polyketide synthase. Clusters containing such a pair of PKS genes have been identified in the biosynthesis of resorcylic acid lactones or dihydroxyphenylacetic acid lactones. *AdPKS7* and *AdPKS8* gene expression patterns correlated with aldaulactone production in different experimental conditions. The present results highly suggest that both genes are responsible for aldaulactone biosynthesis.

## Introduction

Many factors are involved in plant fungal infection and disease development processes. Notably, cell wall degrading enzymes and phytotoxins play a key role in infection and virulence of necrotrophic phytopathogenic fungi^1–3^. Phytotoxins have been divided into two categories based on their specificities^4^: non host-specific toxins (NHSTs), which are the first type of toxins, numerically larger^5^, and host-specific toxins (HSTs). NHSTs can affect a wide range of plant genera with more or less effect and are mainly linked with the intensity of plant disease. HSTs have a toxic effect in a plant genus or species which have a specific target, encoded by a so-called susceptibility gene, which determine whether the fungus can infect the plant or not^1,6,7^. HSTs were discovered mainly in Alternaria and Cochliobolus genera^8–11^. HSTs and NHSTs have various chemical natures, some of them are ribosome synthesis-dependent peptides but most are secondary metabolites (SMs)^1,2,5^.

SMs are divided into four classes depending on their biosynthetic origin: polyketides (PKs), non-ribosomal peptides (NRPs), terpenes and indole alkaloids^12^. Hybrids between those classes also exist. Focusing on the most represented (NRPs, PKs, and their hybrids), their biosynthetic pathways involve enzymes usually encoded by co-regulated genes organized in clusters. In those clusters, one or two core genes encode for NRP-synthases (NRPSs), PK-synthases (PKSs), NRPS-PKSs or PKS-NRPSs responsible for the biosynthesis of the molecule backbone. Then, tailoring enzymes encoded by other genes of the same cluster modify the molecule backbone by functional-group transfer or redox reactions^13^. These clusters also often include genes encoding specific transcription factors, transporters and toxin resistance proteins^13,14^.

Three types of PKS have been described and extensively reviewed in bacteria^15,16^. In fungi, only type I and III are reported. Fungal type III PKSs are poorly known; in facts, only five are experimentally characterized^17^, and few are predicted (between zero and two) in sequenced fungal genomes^17,18^. They are known to synthesize resorcylic acid-type compounds. Most fungal PKSs belong to type I and correspond to large multi-domain and multifunctional enzymes that act iteratively to catalyze the condensation of malonyl-CoA with an acyl-CoA acting as starter^12,13^. Iterative type I PKSs could be subdivided into three types, depending on the degree of reduction in the resulting PK: non-reducing (NR)-, partially-reducing (PR)- and highly-reducing (HR)-PKSs^15,19^. They contain at least a minimal core module of three domains -ketosynthase (KS), acyltransferase (AT) and an acyl carrier protein (ACP) domain- to catalyze head-to-tail Claisen condensation of acetyl-CoA^20,21^. Reducing-PKSs contain optional domains catalyzing the reduction of the β-ketothioester chain, namely ketoreductase (KR), dehydratase (DH), and enoyl reductase (ER) domains. By contrast, NR-PKSs always contain a starter unit ACP transacylase (SAT) domain which catalyzes the transfer of the starter unit to the ACP-domain. NR-PKSs can contain a product template (PT) domain involved in poly-β-ketoester cyclisation^22–25^. NR-PKS can also contains two kind of release domain: a thioesterase (TE) domain or a C-terminal reductase domain (R)^26–29^. The TE domain catalyzes the hydrolysis of the bond between the PK and the enzyme itself and thus releases the resulting PK with or without a cyclization reaction. The R domain catalyzes a polyketide chain release by NAD(P)H-dependent reduction. Other additional domains, such as methyltransferase (MT) domain, were found in both NR-PKS and HR-PKS.

NRPSs are modular megaenzymes, each module being responsible for the addition of a single amino acid to the NRP. One NRPS contains a minimal set of three domains: a condensation (C) domain, an adenylation (A) domain, and a peptidyl carrier protein (PCP) domain^30^. The A domain is responsible for selecting and adenylating a specific amino acid that is then linked by a thioester function to a prosthetic phosphopantetheinyl group on the PCP domain. Substrates of the NRPS A domains extend far beyond the twenty proteinogenic amino acids^31^. At last, the C domain catalyzes an amide bond synthesis between the amino acid and the peptide attached to the PCP domain of the preceding module and so on to the next module. NRP diversity and specificity are mediated through additional tailoring domains, such as epimerase (E) domain and the amino acid specificity of the A-domains. Some fungal NRPS also contain TE- or R-type release domains^29,32^ Hybrids of those enzymes, i.e. combination of PKS and NRPS domains, could be PKS-NRPS or more rarely NRPS-PKS, depending on the orientation of the domains. PKS-NRPS contain HR-PKS and the NRPS set of the three minimal domains, while NRPS-PKS contain the NRPS A, C and PCP domains upstream a KS domain^12,33^.

In this study, we focused on a necrotrophic Dothideomycete fungus, *Alternaria dauci* [(Kükn) Groves & Skolko], the causal agent of *Alternaria* leaf blight on carrot leaves^34^. *Alternaria* leaf blight is the most important aerial disease on carrot world-wide^35,36^. No full carrot resistance against *Alternaria* leaf blight is known, while only partial resistance is observed. Indeed, a strong correlation between carrot plant resistance against *A. dauci* and carrot cell resistance to fungal exudates, and more specifically to the organic extracts, argues for the involvement of toxins in this plant-pathogen interaction^37^.

In the *Alternaria* genus, the toxin roles in the pathogenicity are particularly well described with more than seventy toxins identified among pathogenic species^38^. Remarkably, numerous phytopathogenic *Alternaria* species produce HSTs that cause high specificity for their host^8^. So far at least seven phytotoxins have been isolated and characterized from *A. dauci* cultures: zinniol, alternariol, alternariol monomethyl-ether, *α*-acetylorcinol, *p*-hydroxybenzoic acid and aldaulactone^39–43^. To our knowledge, little is known about their biosynthetic pathways, except for the alternariol biosynthetic cluster that was recently predicted by sequence homology^44^. The contribution of these toxins to *A. dauci* pathogenicity has been shown only for zinniol and aldaulactone^39,40^. However, recent studies highlighted that only high concentrations of zinniol were toxic to carrot cells^37,45^. On the contrary, in vitro carrot cell toxicity of aldaulactone was pointed out at concentrations observed in *A. dauci* cultures. In an original way, this toxicity is negatively correlated with carrot cell resistance^39^. Interestingly, it is only the second evidence, with SS-toxin in the *Stemphylium solani*-*Allium sativum* pathosystem, of a link between fungal SMs and partial resistance^37,39,46^. Aldaulactone seems to be the key component of this link in the *A. dauci-D. carota* pathosystem, although it was shown that other toxins might be involved in *A. dauci* pathogenicity^39^.

Unlike the SS-toxin, the chemical structure of aldaulactone has been described: it is a benzenediol lactones. PK benzenediol lactones aggregate two molecule families containing a 1,3-benzenediol moiety connected with a macrolactone: the dihydroxyphenylacetic acid lactones (DALs) and the resorcylic acid lactones (RALs). For a few DALs and RALs (zearalenone, hypothemycin, radicicol, 10,11-dehydrocurvularin)^47–53^, the genetic and molecular bases of their biosynthetic pathways are known in fungi. The same particular type I HR-PKS-NR-PKS collaboration pattern for the biosynthesis of the molecular backbone is observed in all those pathways. The HR-PKS catalyzes the biosynthesis of a highly reduced aliphatic PK used as a substrate by the NR-PKS to catalyze a non-reduced elongation. Afterward, the PT domain of the NR-PKS catalyzes the formation of a 1,3-benzenediol ring. The PT domain determines the regioselectivity of the cyclisation. A C2-C7 aldol or a C8-C3 aldol condensation produces respectively a RAL or a DAL^48^. Finally, the TE domain catalyzes the macrolactone formation^29,54^.

The first aim of this study was to provide an appraisal of PKS- and NRPS-encoding gene diversity in *A. dauci* and their repartition within the *Alternaria* genus. In order to do so, we obtained the first reported sequencing of the *A. dauci* transcriptome. We jointly used an improvement (N50 = 4.493 Mb) of the previously published genome assembly (N50 = 13 kb)^55^ and that transcriptome to identify potential SM clusters. We found 18 PKS, 6 NRPS and 1 PKS-NRPS-encoding genes. We also predicted SM genes in a set of 20 other genomes among phytopathogenic *Alternaria* species. Among the predicted PKS- and NRPS-encoding genes, we found some genes involved in already known biosynthetic pathways but also a majority of newly described genes. Our analyses allowed to identify 5 *A. dauci-*specific SM genes, underlying *A. dauci*’s potential to produce yet-to-be described SM, including toxins.

Our second aim was to better understand the biological activity and biosynthesis pathway of aldaulactone. We first checked aldaulactone leaf toxicity on *Nicotiana benthamiana* (order: *Solanales)*, a distant species from *D. carota* (order: *Apiales*). We then hypothesize that aldaulactone biosynthesis follows the same pattern as for other benzenediol lactones, and involves a HR-PKS and a NR-PKS. Based on our knowledge of RALs and DALs biosynthesis, we identified a candidate cluster for the aldaulactone biosynthesis pathway by homology search, phylogenetic and in silico retro-biosynthesis approaches^52^. Furthermore, we pointed out a significant correlation between aldaulactone production and the expression level of the HR-PKS (*AdPKS7*) and the NR-PKS (*AdPKS8*) genes belonging to this candidate cluster. Our data indicates that the cluster containing *AdPKS7* and *AdPKS8* may be responsible for aldaulactone biosynthesis. Moreover, our results further validate the potential to discover novel toxins involved in both *A. dauci* pathogenicity and *D. carota* partial resistance.

## Results

### Aldaulactone phytotoxicity test on tobacco

**Figure 1:**
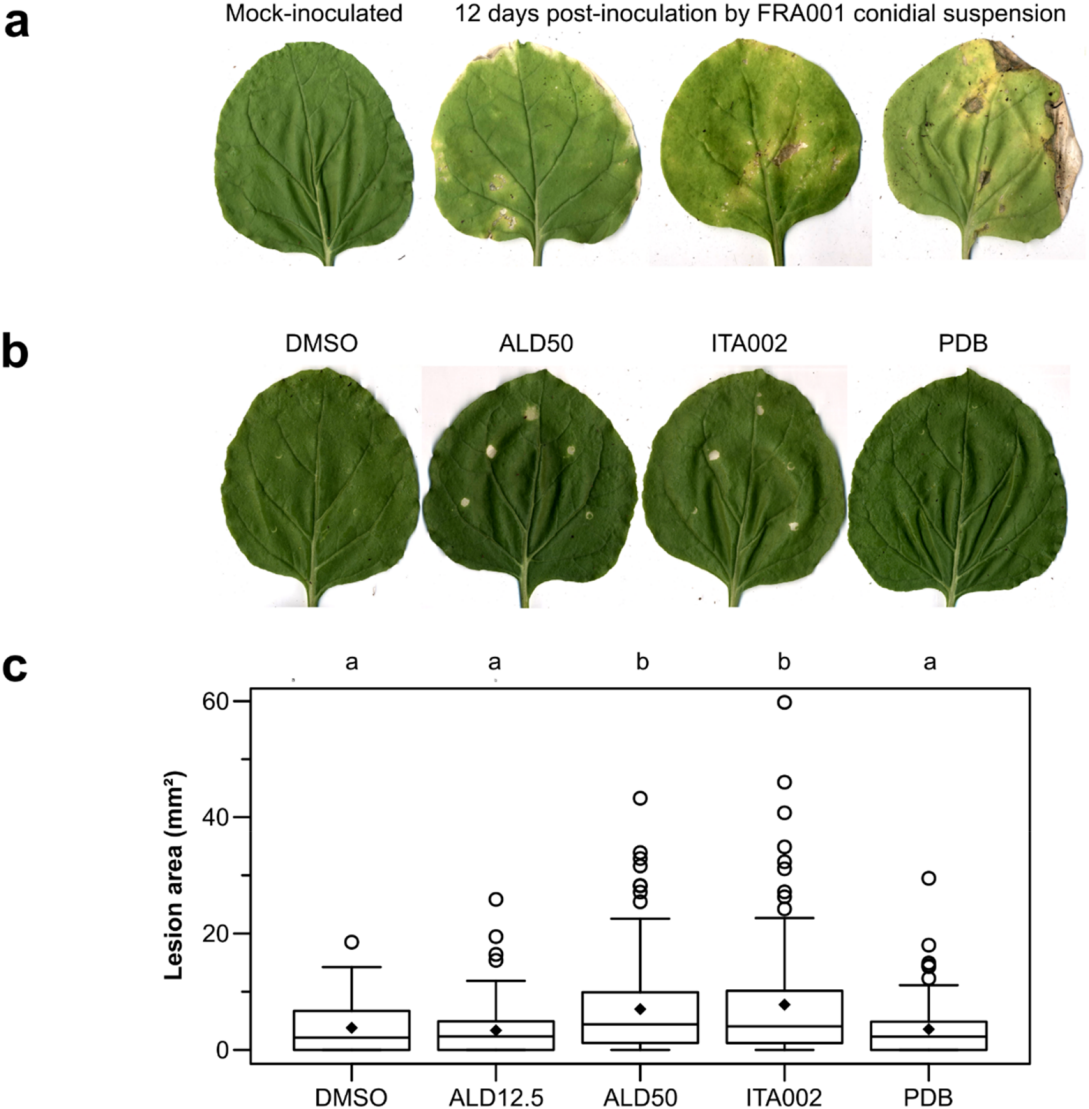
(**a**) *N. benthamiana* leaves 12 days post-inoculation by spreading FRA001 *A. dauci* strain conidial suspension with a paint brush. (**b**) *N. benthamiana* leaves 9 days after infiltration with various solutions. DMSO: 0.1% DMSO diluted in ultrapure water; ALD50: Aldaulactone at 50 µg mL^−1^ diluted in 0.1% DMSO solution; ITA002: organic phase of culture filtrate from ITA002 strain grown in PDB medium and diluted in 0.1% DMSO solution. (**c**) Lesion area distributions. Same legends as in B/ excepting ALD12.5: Aldaulactone at 12.5 µg mL^−1^ in 0.1% DMSO. Each box plot represents area distribution amongst 45 infiltrations (three independent repetition of 15 infiltration dots on 3 different plants). Diamonds represent the means. Letters represent the result of a Siegel and Castellan *post-hoc* test realized after a Kruskal–Wallis test (*p* value = 2546.10^−6^).

In our previous paper^39^, only in vitro proofs were provided for aldaulactone toxicity. Direct *in planta* evidence of aldaulactone toxicity was not yet established, since carrot leaf infiltration with the toxin is challenging, because of leaf fragility (results not shown). Alternatively, experiments of plant infection or infiltration were performed using the *N. benthamiana* model. Necrotic leaf lesions (black spots surrounded by a yellow halo) on tobacco were obtained after inoculation with a conidial suspension of FRA001 strain (Fig. 1a). Isolation from symptomatic leaf pieces allowed us to obtain fungal colonies producing conidia exhibiting a typical *A. dauci* morphology. From those isolates, species identification was done by sequencing portions of three target genes (*ITS, EF1-α, IGS*). The obtained sequences exactly matched those of *A. dauci* strain FRA001 (data not shown). These results showed that *A. dauci* is pathogenic on tobacco in our experimental conditions.

Meanwhile, phytotoxicity tests were performed by leaf infiltration using two aldaulactone concentrations and an organic extract of ITA002 culture medium (Fig. 1b,c). Lesion areas nine days post-infiltration of the control conditions (0.1% DMSO and PDB) were in the same statistic class than the ones observed after infiltration using 12.5 µg mL^−1^ aldaulactone. The lesion areas produced by the infiltration with 50 µg mL^−1^ aldaulactone or by the ITA002 organic extract were significantly higher than control conditions. Aldaulactone is able to produce necrotic lesions on tobacco leaves.

### Improvement in *Alternaria dauci* genome assembly by RNA-seq-mediated and reference genome scaffolding.

**Figure 2:**
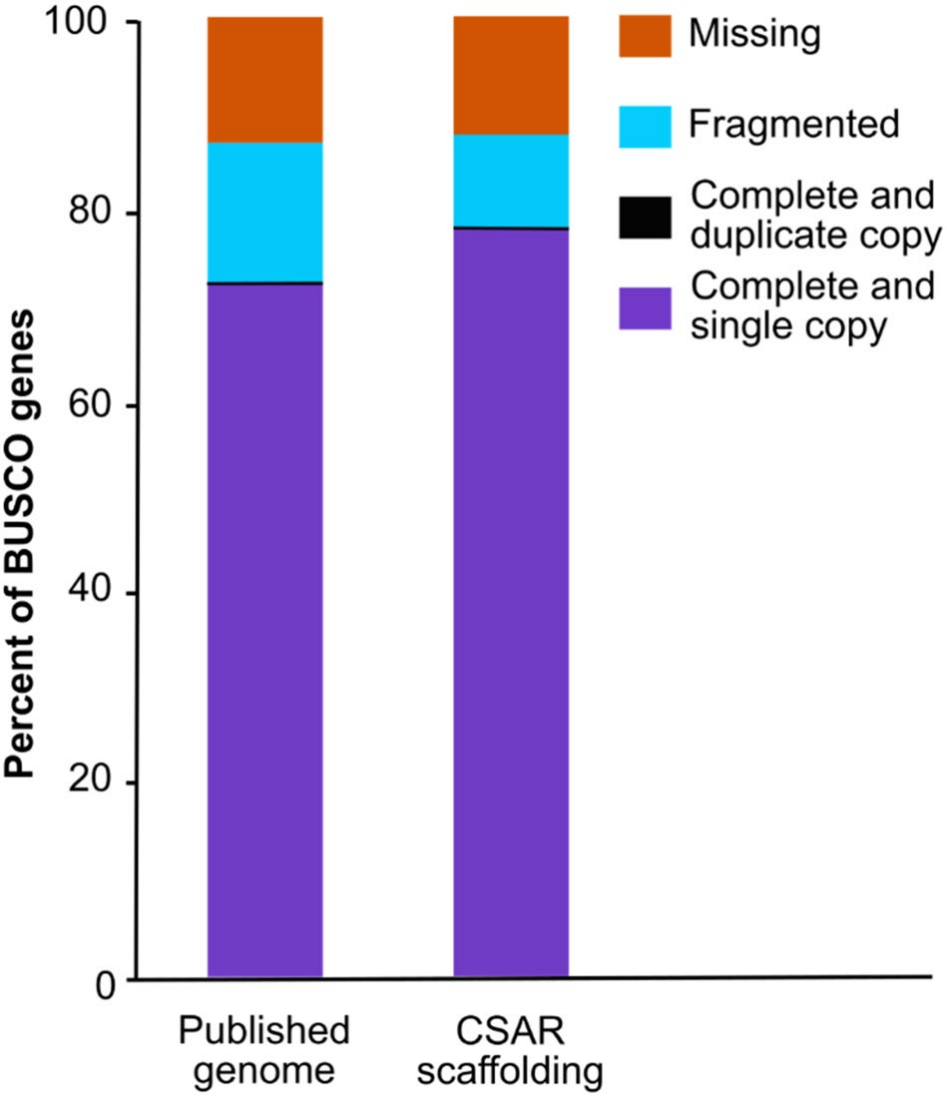
BUSCO (Benchmarking set of Universal Single-Copy Orthologues) analysis of two *A. dauci* genome assembly. Percentage of completeness was based on the Pezizomycotina gene dataset of BUSCO. Published genome: Genome of *A. dauci* strain BMP0167 available on *Alternaria* genome database^55^. CSAR scaffolding: assembly of the published genome helped by FRA001 transcriptome data obtained in this study.

The assembly of the only *A. dauci* genome published on a database is composed of many small contigs (N50 = 13,282 bp; number of contigs = 12,030), shorter than the typical length of SM gene clusters, that amount to several tens of kilobases^55^ (Table 1). Moreover, *A. dauci* genome assembly contained only 72.2% Pezizomycotina BUSCOs genes with 14.5% fragmented and 13.2% missing data (Fig. 2). Two complementary scaffolding strategies were performed to improve the quality of the assembly: (1) genome assembly improvement using the AGOUTI tool^56^ with the RNA sequencing data from FRA001 strain (available at http://www.ncbi.nlm.nih.gov/bioproject/790446), and (2) reference-based (*A. solani* genome^57^) genome re-assembly with the CSAR tool^58^. After applying the first strategy, the number of contigs/scaffolds (> 100 bp) decreased from 4,010 to 3,139 and N50 increased from 13,282 to 18,857 bp (Table 1). Due to the method, the assembly improvement was expected only in the expressed areas of the genome, explaining the apparently modest gains made. Nevertheless, many contigs containing SM cluster were reassembled.

**Table 1:** Assembly summary statistics applied to the different assemblies of *Alternaria dauci* genome data set.

Assembly	Published genome^55^	AGOUTI re-assembly	CSAR scaffolding
No. of contigs/scaffolds	12,030	11,056	7594
No. of contigs/scaffolds (> 100 bp)	4010	3139	553
N50 (bp)	13,282	18,857	4,493,962
L50	736	507	3
Maximal length (> 100 bp)	73,673	104,835	6,771,279
Genome size (Mb)	32.1	33.1	33.4
GC (%)	51.61	51.61	50.68
No. of N per 100 kbp	83.19	3157.84	4021.31
No. of predicted genes	11,981	12,388	12,939

AGOUTI re-assembly: re-assembly by the AGOUTI tool^56^ of the published genome^51^ helped by FRA001 transcriptome data obtained in this study. CSAR scaffolding: reference-based (*A. solani* genome^57^) genome re-assembly with the CSAR tool^58^. N50: length of the shortest contig/scaffold in the smallest subset of contigs whose length sum makes up half of genome size. L50 number of contigs in that subset. GC (%): guanine-cytosine content.

The second scaffolding strategy gave a new improved assembly called below “CSAR genome assembly”. When applying the second scaffolding strategy, a strong improvement in genome contiguity was obtained with a decrease from 3139 to 553 scaffolds (> 100 bp) and a N50 increase from 18,857 bp to 4.49 Mpb (Table 1). The BUSCO completeness of 78.1% with 9.6% fragmented and 12.2% missing data was largely improved over the 72.2% of the published genome assembly even if the percentage remain still relatively low (Fig. 2). This scaffolding therefore improved genome contiguity and gene completeness, and produced an assembly including 7594 scaffolds with a total genome size of 33.4 Mb and a GC content of 50.68% (Table 1).

The RNA-Seq library was constructed from FRA001 strain and sequenced using Illumina HiSeq2000: 161.96 million of paired-end reads and 160.5 million of cleaned reads were obtained. Reads mapping on CSAR genome assembly was performed by HISAT2. A total of 87.8% of those reads aligned concordantly one time. After this step, StringTie and Cufflinks were used to assemble the RNA-Seq alignments into 12,939 transcripts (Table 1). The unigene N50 was 1713 bp consistent for a fungal transcriptome.

### Bioinformatic prediction of putative SM gene clusters in *A. dauci*

**Figure 3:**
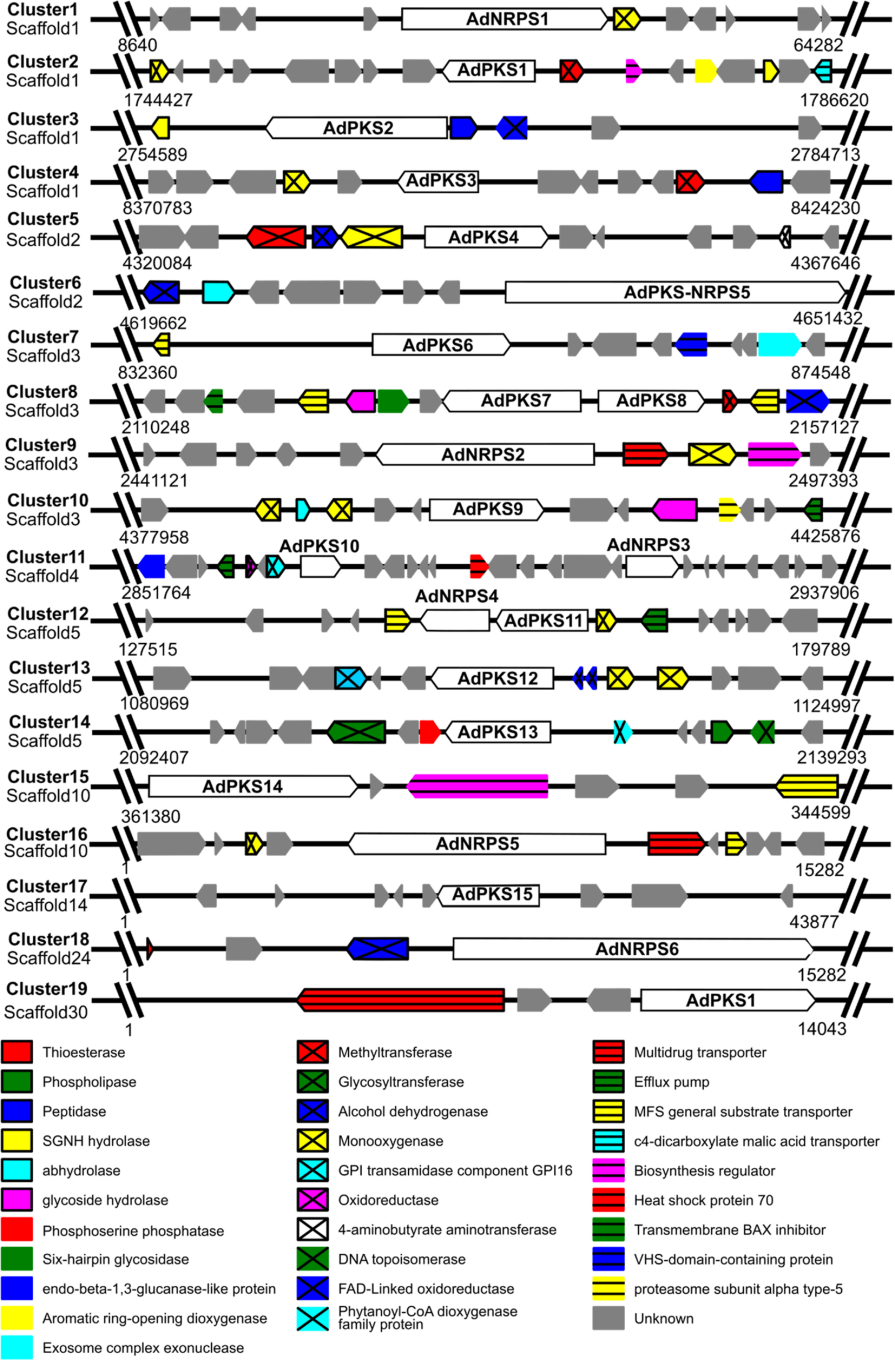
*Alternaria dauci* secondary metabolism cluster organization as predicted by antiSMASH and SMURF. BLASTP searches were performed to identify a potential function for each gene in the cluster (more details in Supplementary Table S1).

A survey of the *A. dauci* genome and transcriptome dataset for SM genes was performed. BLASTP analysis of KS and AT domains on the published genome^55^ predicted 20 PKS-like encoding sequences (named sequence 1-20), most of them truncated. Predictions mediated by AntiSMASH and SMURF on the CSAR genome re-assembly allowed to predict 19 clusters including 20 PKS and NRPS genes, 2 partial PKS genes (Fig. 3, Supplementary Table S1).

**Figure 4:**
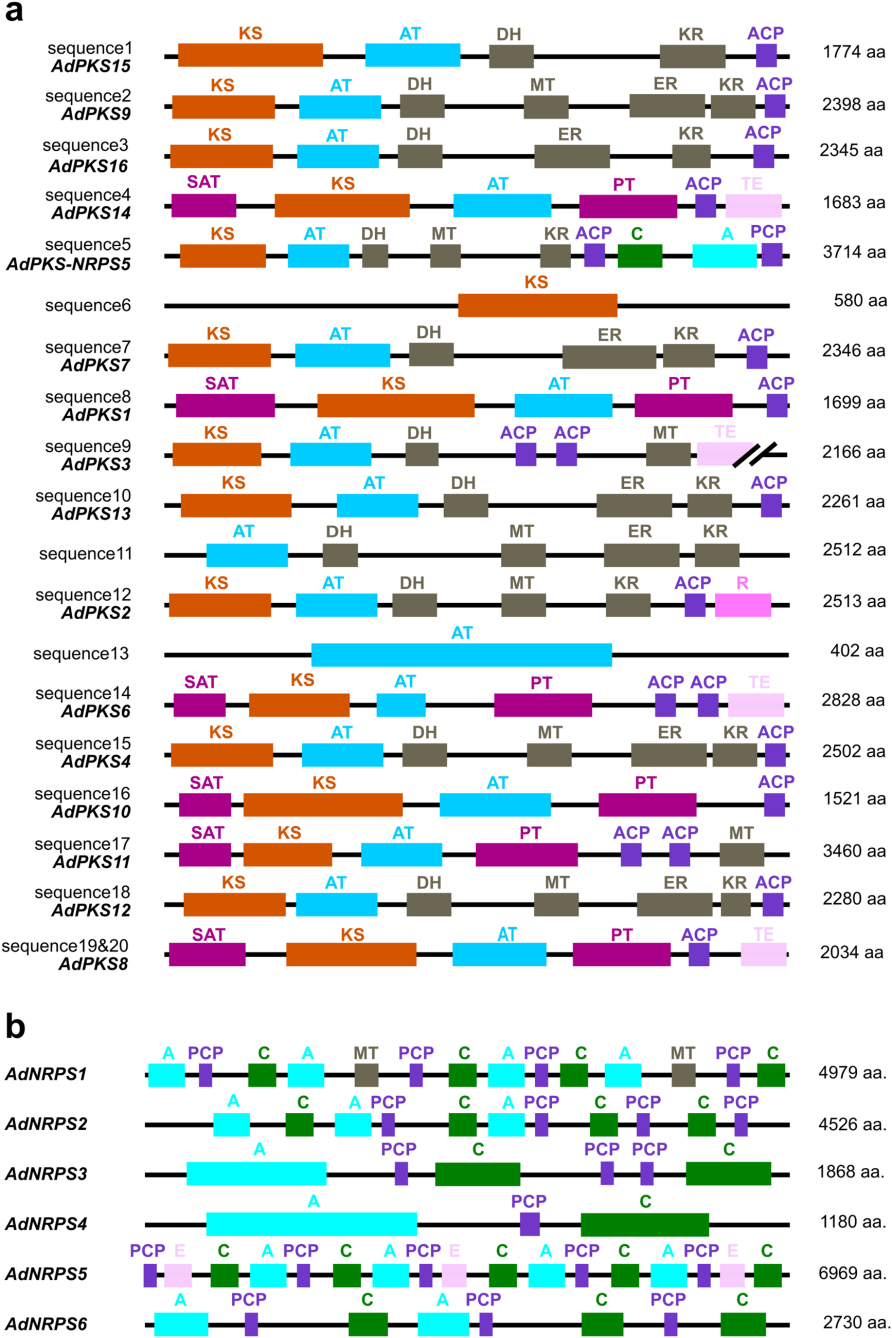
Domain organization of the PKS, PKS-NRPS hybrid and NRPS proteins predicted in the *A. dauci* genome. (**a**) PKS and PKS-NRPS hybrid proteins, (**b**) NRPS proteins. Abbreviations are as following: A: adenylation domain; ACP: acyl carrier protein; AT: acyltransferase; C: condensation domain; DH: dehydratase; E: epimerization; ER: enoyreductase; KS: ketosynthase; KR: ketoreductase; MT: methyltransferase; PCP: peptidyl carrier protein; PT: product template; SAT: starter unit ACP transacyclase; R: reductase; TE: thioesterase. Gene names are given in bold italics.

Sequences 6, 11, 13, 19 and 20 did not show the 3 minimal domains (KS-AT-ACP; Fig. 4a). Sequences 11 and 13 were only predicted in the first genome annotation. Sequences 6 and 13 were consistent with a single domain. We hypothesized that these 2 sequences do not correspond to PKS genes. For sequence 3 (KS-AT-DH) and 11 (AT-DH-ER-KR), misassembly could cause the missing domains or these two sequences may correspond to pseudogenes. Sequence 19 (SAT-partial KS) was localized in cluster 8 in scaffold 3 and took end in a sequencing gap of the genome. Sequence 20, encoding a NR-PKS 3’-end (AT-PT-ACP-TE), was consistent with the whole scaffold 116 and was found to be expressed. Primers designed in sequence 20 and on both sides of the scaffold 3 gap next to sequence 19 led to a positive PCR amplification and subsequent sequencing of a 1308 bp amplicon. Sequences 19 and 20 are part of a single gene, named AdPKS8, and scaffold 116 corresponds to the gap near sequence 19 in scaffold 3. All SM cluster and domain predictions were performed again using this combined sequence integrated in the genome reassembly and reported in Figs. 3 and 4. Among all those SM genes, AdPKS1, 3, 6, 7, 8, 10, 13, 16 and AdNPRS1, 2, 3 were found in our *A. dauci* transcriptome.

To study the putative functions of those predicted proteins, homologies with characterized proteins were pointed out (Supplementary Tables S1, S2). *A. alternata* TES (A0A144KPJ6.1) and TES1 (A0A144KPK9.1), two enzymes responsible for tentoxin biosynthesis, were found to have more than 80% identity with AdNRPS1 and a protein encoded by another cluster 1 gene. AdNRPS2 and AdNPRS3 are linked to siderophore-mediated iron metabolism. Indeed, AdNRPS2 in cluster 9 showed 59% identity with *Bipolaris maydis* intracellular siderophore synthetase (Q5D6D7.2), whileAdNRPS3 in cluster 1 exhibited 86% similarity with *Bipolaris oryzae* extracellular siderophore synthase (Q09MP5.1)^59^. AdPKS6 in cluster 7 showed 95% similarity with *A. alternata* melanin synthase (BAK64048.1). As previously described^44^, *A. dauci* genome contains a homologue of *A. solani* alternariol synthase cluster (cluster 2). AdPKS12 in cluster 13 showed 91% identity with *A. solani* alternapyrone synthase (Q5KTM9.1) and the whole cluster was conserved. AdPKS13 in cluster 14 had 91% identity with *A. solani* aslaniol synthase (Q2ABP6.1). At last, AdPKS14 in cluster 15 presented 95% identity with *Alternaria cinerariae* Dhc5 (dehydrocurvularin biosynthesis protein 5, KT271474.1). No homologue to Dhc3, the other PKS necessary for dehydrocurvularin biosynthesis (KT271472.1) was found in the *A. dauci* genome.

### Diversity and repartition of SM genes in the *Alternaria* genus

**Figure 5:**
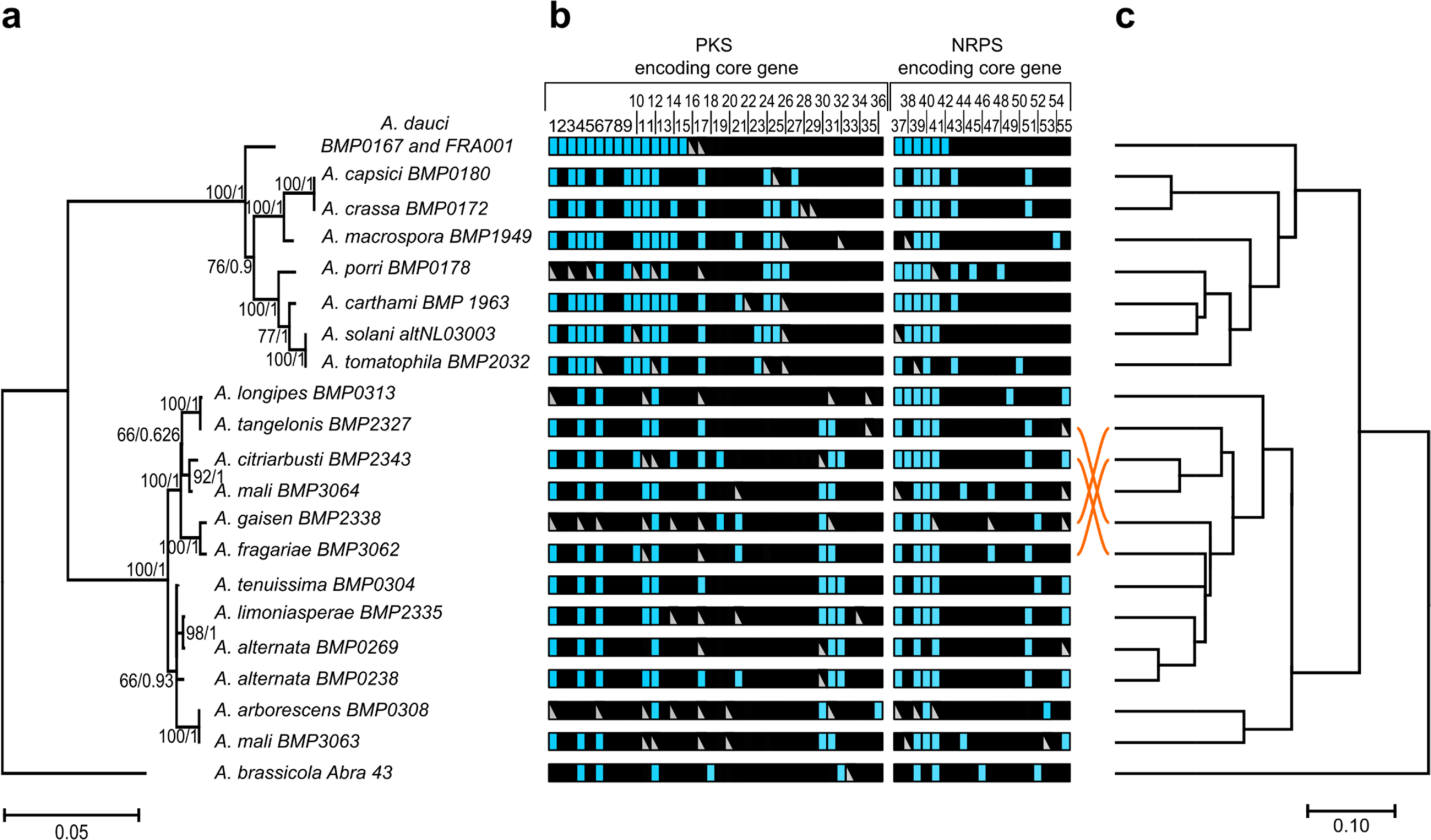
Phylogenetic tree and distribution of *NRPS*, *PKS* and hybrid core genes among *Alternaria* species based on antiSMASH SM gene prediction from genomic sequences and Tblastn analyzes. (**a**) *Alternaria* species phylogenetic tree. The phylogenetic tree was obtained by ML under the GTR + GAMMA substitution model with a bootstrapping of 1000 replicates from an alignment of four concatenated housekeeping gene sequences. To generate Bayesian probability values, Bayesian analyses were performed with the MrBayes algorithm. (**b**) Distribution of *NRPS*, *PKS* and hybrid core genes within each species. Blue box: gene is present. Genes present in the same column share more than 80% identity and are considered orthologues. Gray triangle: a partial copy or a pseudogene is present. Black box: gene was not found. (**c**) UPGMA dendrogram generated from a binary matrix of SM core gene presence and absence derived from (**b**) with the use of Jaccard coefficients to compare between sets of variables. This dendrograms follows closely the phylogenetic tree shown in (**a**) except for four species only (orange streaks).

To compare *A. dauci* potential ability to produce SMs with other *Alternaria*, SM core genes were predicted by antiSMASH from 20 *Alternaria* genomes and manually curated. Predicted proteins with at least 80% of the total length exhibiting 80% identity were grouped and numbered. Fifty-five such gene groups, including 34 PKSs, 2 PKS-NRPSs, 2 NRPS-PKSs and 17 NRPSs, were predicted (Fig. 5, Supplementary Table S2). The SM gene presence/absence patterns showed that 18 SM core genes were private to a single genome, with *AdPKS2*, *AdPKS7, AdPKS8, AdPKS16* and *AdNRPS6* private to the *A. dauci* genome. Conversely, *AdPKS6* or *AdPKS12* were present in all examined *Alternaria* genomes. Remarkably, *AdPKS1*, *AdPKS13* and sequence 11 were present in all *Alternaria* genomes studied, excepting *A. brassicicola.* The other SM genes exhibited mosaic patterns of distribution through *Alternaria* genomes. Globally, *Alternaria* genomes contained 6–18 *PKS* genes, 4–9 *NRPS* genes, 0 or 1 hybrid *PKS-NRPS* and 0 or 1 *NRPS-PKS* per genome. Interestingly, *Alternaria* genomes of the *porri* section contained more genes encoding for PKS than those of the *alternata* section and even more than *A. brassicicola*. To support this observation, a dendrogram was produced from a gene presence/absence matrix and compared with the phylogeny of the studied *Alternaria* strains (Fig. 5c). This allowed to differentiate most species, excepting *A. tangelonis* and *A. tenuissima,* while this phylogenetic analysis also failed to distinguish *A. tenuissima* and *A. alternata*. Both dendrograms showed a similar strain repartition in three clades: (1) the *porri* section strains, (2) the *alternata* section strains, and (3) *A. brassicicola*. Moreover, strain distribution within each clade was strongly similar in both dendrograms.

### Phylogeny of KS and PT domain of *A. dauci* PKS

**Figure 6:**
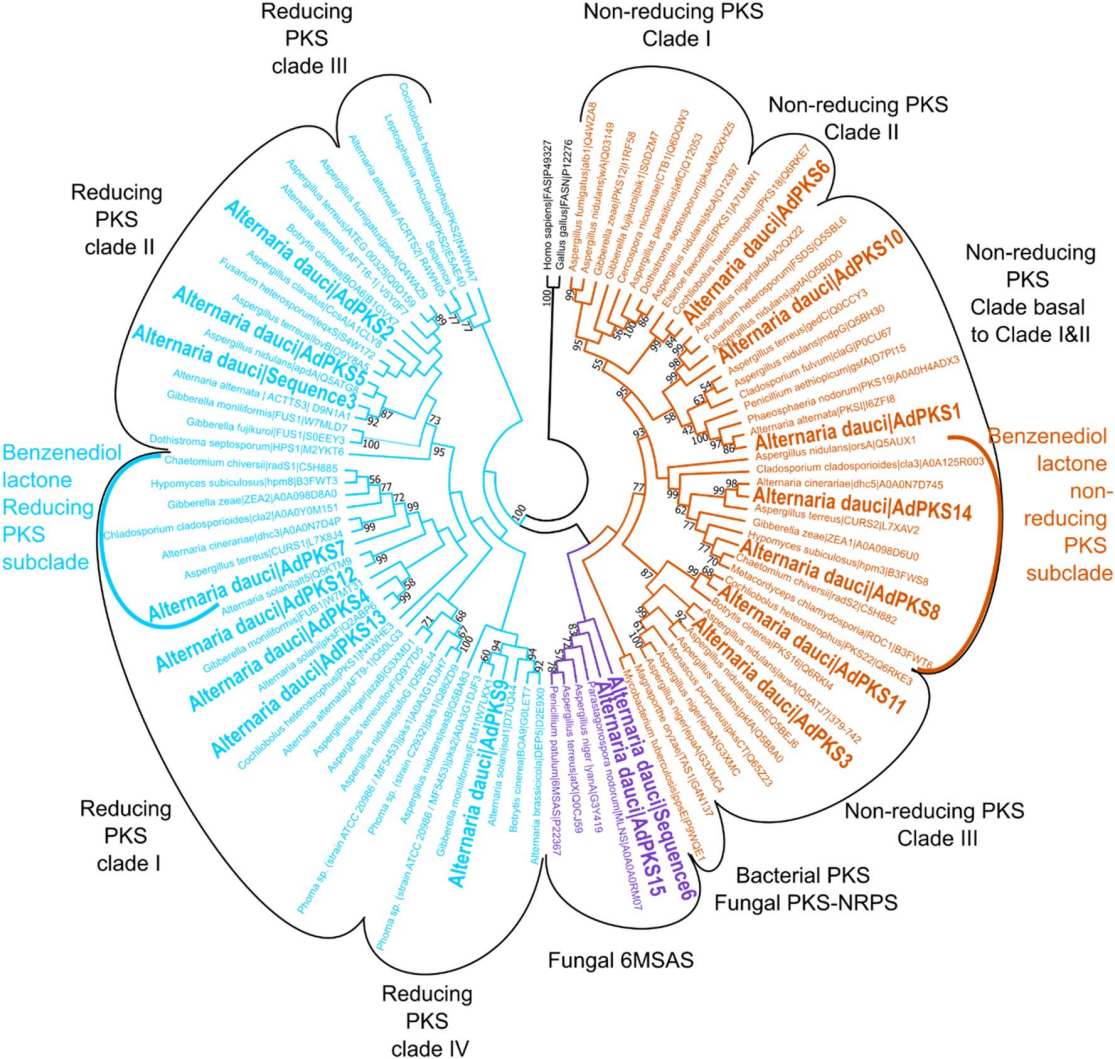
Phylogenetic analysis of the KS domain of all the Dothideomycetes PKS sequences characterized and reviewed in Uniprot database and the *A. dauci* predicted PKSs (in bold). The phylogenic tree was inferred using maximum parsimony method with tree-bisection—regrafting algorithm and 1000 replicates to calculate bootstrap values. Clades indicated in black were named according to^60^.

A phylogenetic study of the KS and PT domains was performed to classify the AdPKS sequences among characterized PKSs from Dothideomycetes and to find candidates for aldaulactone biosynthesis, i.e. PKSs homologous to NR-PKS and HR-PKS involved in DAL biosynthesis. Eighty-two Dothideomycete KS-sequences from^60^ and from the UniProt reviewed database were used for a maximum of parsimony tree reconstruction (Fig. 6, Supplementary Table S3).

The clades produced and AdPKS-domain structures predicted were consistent to those previously described^60^. HR-PKS were divided in four clades: HR-PKS clade I (KS-AT-DH-(MT)-ER-KR-ACP), HR-PKS clade II (KS-AT-DH-(MT)-KR-ACP-(C)-(A)-(PCP)), HR-PKS clade III (KS-AT-DH-ER-KR-PP-(PP)), and HR-PKS clade IV (KS-AT-DH-MT-ER-KR-ACP). NR-PKS were divided in four clades: a clade basal to clades I and II ((SAT)-KS-AT-PT-ACP-(ACP)-(TE)), NR-PKS clade II (SAT-KS-AT-PT-ACP-ACP-TE), NR-PKS clade II (SAT-KS-AT-PT-ACP-ACP-TE) and NR-PKS clade III ((SAT)-KS-AT-ACP-(ACP)-MT-(TE)). Two additional basal clades were found, corresponding to the PR-PKSs clade (6-MSAS; KS-AT-DH-KR-ACP) and the other one corresponding to both fungal PKS-NRPSs and the bacterial PKSs.

Interestingly, HR-PKS clade I contained a very well defined subclade (bootstrap value of 99%) that regrouped AdPKS7 and all HR-PKS involved in benzenediol lactone biosynthesis. AdPKS4, AdPKS12 and AdPKS7, clearly belonged within HR-PKS clade I, but not in the same subclade as AdPKS7. AdPKS2, AdPKS5 and sequence 3 clustered in HR-PKS clade II. AdPKS9 clustered within the HR-PKS clade IV. AdPKS15 and the sequence 6 clustered in the PR-PKSs clade. The NR-PKS clade basal to clade I&II contained two monophyletic subclades, one including AdPKS1 and AdPKS10, the other gathering AdPKS8, AdPKS14 and NR-PKSs involved in benzenediol lactone biosynthesis. NR-PKS clade II included AdPKS6. AdPKS3 and AdPKS11 clustered in NR-PKS in clade III. According to KS-domain phylogeny, AdPKS7 and AdPKS8 or AdPKS14 were consistent candidates for aldaulactone biosynthesis.

**Figure 7:**
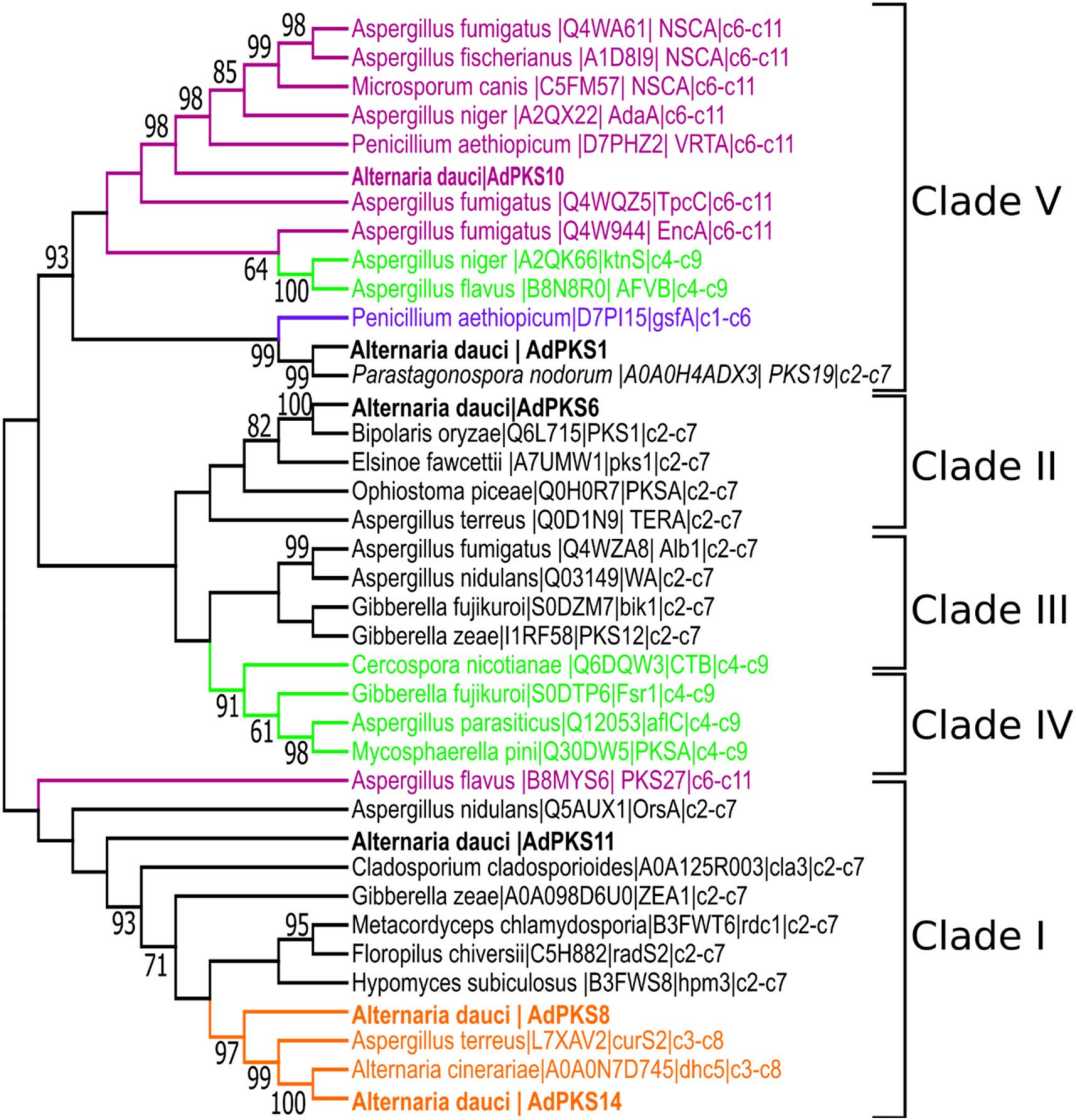
Phylogenetic tree of PT domains of 38 selected NR-PKS sequences. The regioselectivities catalyzed by PT domain are indicated when known. Phylogenetic analysis was conducted using the bootstrap (1000) maximum parsimony method. PKSs were grouped in five monophyletic clades consistent to those described by^27^.

The phylogenetic tree of PT domains was constructed from 35 NR-PKSs, including 6 AdPKSs, to study first-ring aldol-cyclization stereoselectivity (Fig. 7). PKSs were grouped in 5 monophyletic clades consistent to those described by^27^. AdPKS6 PT domain clustered within clade II. AdPKS1 and AdPKS10 clustered within clade V, respectively with C2–C7 and C6–C11 type PT domains. Clade I contained two monophyletic subclades, the first one clustered C2–C7 type PT domains, the second one C3–C8 type PT domains. AdPKS11 belongs to the first subclade, AdPKS14 and AdPKS8 to the second one. These results strengthened the fact that AdPKS14 and AdPKS8 are good candidates for aldaulactone biosynthesis.

### Functional analysis of AdPKS for aldaulactone production

**Figure 8:**
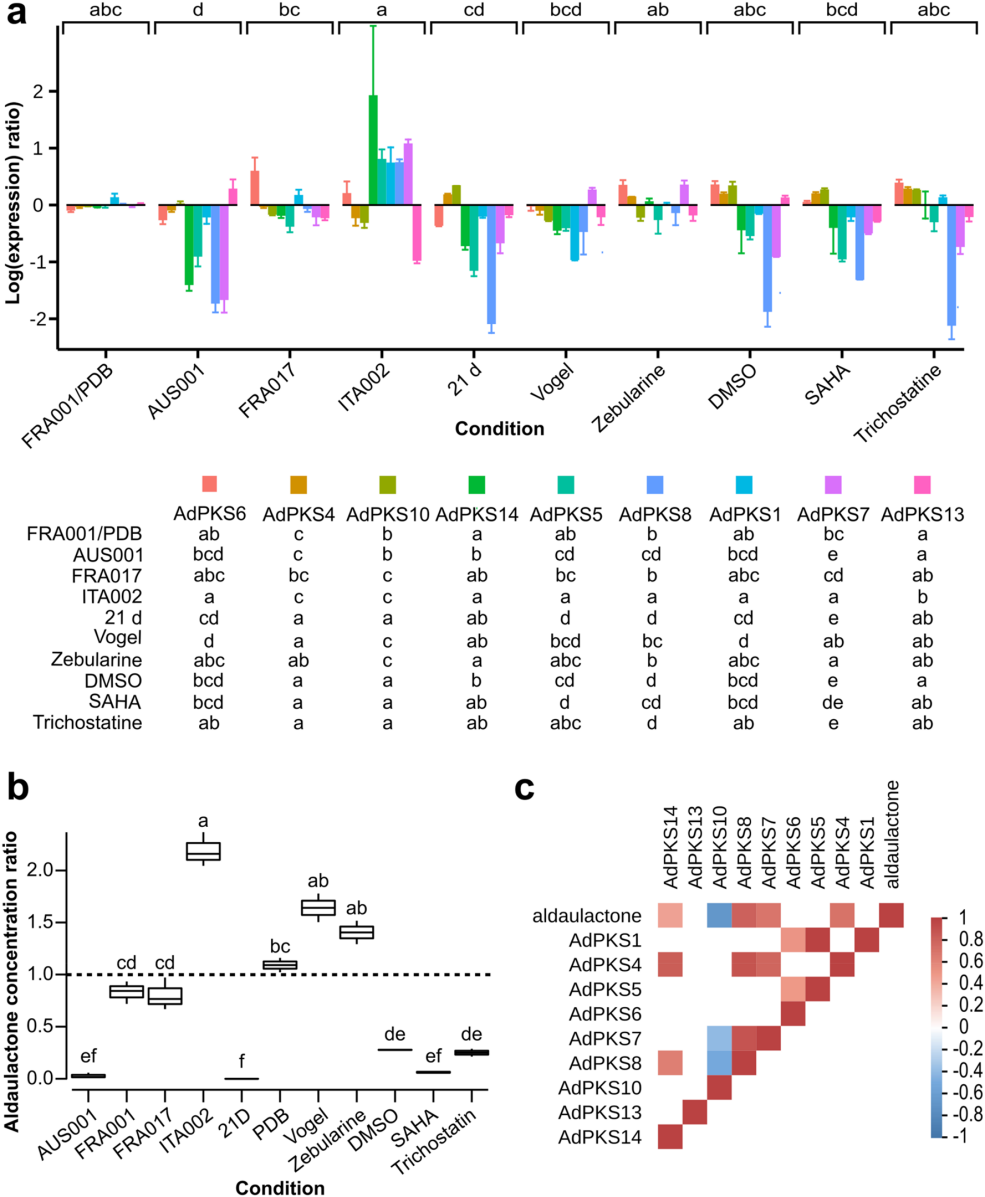
Relative expression of *AdPKS* genes and Aldaulactone production. (**a**) Relative expression of *AdPKS* genes by *A. dauci*. RT-qPCR was performed on RNA from 60-h-old mycelium from different strains and cultivated in various conditions. Four *A. dauci* strains (AUS001, FRA001, FRA017 and ITA002) were grown in PDB medium. FRA001 was also grown in different conditions: 21 days in PDB medium without shaking (21D), minimal medium (Vogel), minimal medium with DMSO (DMSO), or minimal medium with chemical inhibitors of epigenetic regulation (Zebularine, SAHA, Trichostatin) in DMSO. The relative expression of *AdPKS1, AdPKS4, AdPKS-NRPS5, AdPKS6, AdPKS7, AdPKS8, AdPKS10, AdPKS14* were studied. The expression ratio was calculated with FRA001 grown in PDB as a control sample and normalized thanks to mean expression of three housekeeping genes (*EF1*, *actin* and *G3P*). Data presented are the averages on at least two biological replicates and three technical replicates; the error bars represent standard errors. Data were analyzed by Kruskall-Wallis test (*p* < 0.5) followed by a post-hoc test using the Fisher’s least significant difference with Bonferroni correction (culture modalities or culture modalities and gene as factors). Different letters indicate significant differences. Letters above the figure correspond to culture modalities for all genes. Letters bellow the figure correspond to culture modalities for each gene. (**b**) Aldaulactone yield for *A. dauci* strains in various culture conditions. From the same *A. dauci* cultures as in A., aldaulactone absolute quantification was performed from an organic extract of the culture medium by HPLC–DAD with a standard curve. A ratio was calculated for all the quantifications relative to the mean quantity of aldaulactone in FRA001 60 h cultures in PDB medium. (**c**) Correlation matrix of *AdPKS* gene expression ratio and aldaulactone yield ratio presented in (**a**) and (**b**). Spearman coefficient was calculated. Only significant correlations are shown, if non-significant they appear blank. Color in each cell represents the value of correlation coefficients.

In order to decipher aldaulactone biosynthetic pathway, the correlation between expression levels of 8 *AdPKS* genes and aldaulactone accumulation was investigated by HPLC–DAD and RT-qPCR experiments (Fig. 8). To produce variations in aldaulactone production and *AdPKS* genes expression, the experimental conditions consisted of (1) 4 *A. dauci* strains grown in PDB medium, and (2) *A. dauci* FRA001 strain grown in different conditions. The FRA001 strain grown in PDB medium was taken as a reference and all results are expressed as ratios.

In the same trends as in^39^, FRA017 and FRA001 strains had a same order of aldaulactone production, while AUS001 produced about 30-fold less and ITA002 strain produced between twice and thrice more aldaulactone than FRA001 strain. Considering FRA001 strain, a non-statistically significant higher production of aldaulactone was found in minimal medium (Vogel) and in PDB with zebularine, by comparison with PDB. The addition of DMSO in PDB significantly decreased aldaulactone production, while addition of SAHA or trichostatin had no significant effect (when compared with DMSO). Aldaulactone concentration was below the limit of detection when FRA001 was grown in PDB, without medium agitation during 21 days.

The expression ratios of the 8 *AdPKS* genes in the different experimental conditions presented various patterns. *AdPKS10* expression was repressed in ITA002 strain compared to FRA001, FRA017 and AUS001 strains. Interestingly, for the other *AdPKS* genes studied, the opposite pattern was observed: transcription levels seemed to increase with strain aggressiveness, AUS001 being the less aggressive and ITA002 the more aggressive as reported in^61^.

The Spearman correlation coefficient between ratios of *AdPKS* expression and aldaulactone yields were calculated and 5 of them were statistically significant (*p* < 0.01; Fig. 8c). Among them, the one obtained for *AdPKS10* was negative, while others were positive. The correlation coefficient obtained for *AdPKS14* (r = 0.46) was almost twice weaker than those obtained for *AdPKS-NRPS5*, *AdPKS7* and *AdPKS8* genes (r ≥ 0.81). Furthermore, *AdPKS-NRPS5*, *AdPKS7* and *AdPKS8* expressions were cross correlated.

## Discussion

**Figure 9:**
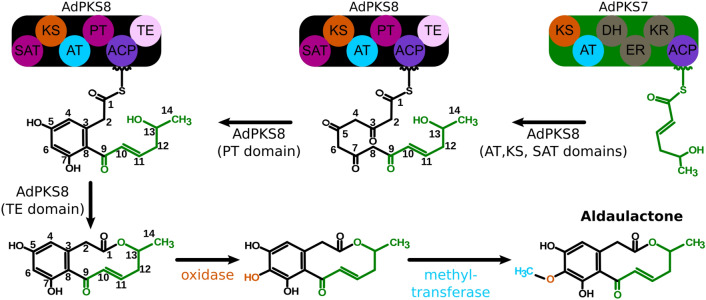
Proposed biosynthetic pathway of aldaulactone in *A. dauci*. The HR-PKS (AdPKS7) produces a reduced triketide. This triketide becomes the starter unit of the NR-PKS (AdPKS8). AdPKS8 catalyzes the addition of four more malonyle-CoA units. The AdPKS8 PT domain catalyzes a C3–C8 aldol condensation. AdPKS8 TE domain then catalyzes the macrolactone cyclisation. Afterward, the cluster 8 oxidase catalyzes the addition of an alcohol function on C6 of the benzene diol. Finally, the methylation of the C6 alcohol function is catalyzed by the cluster 8 methyl transferase.

*A. dauci* belongs to the *Dothideomycetes*, which are known to produce various SM. More than 250 SM have been described from *Alternaria* genus members, a lot of them being HST or NHST^1,62^*.* Interestingly, strong evidence of toxins’ role in *A. dauci* aggressiveness and *D. carota* partial resistance were provided^37^. Among the few toxins investigated in *A. dauci* (zinniol, alternariol, alternariol monomethyl ether…)^39–43^*,* aldaulactone was supposed to explain most, but not all, in vitro toxicity of *A. dauci* exudates. However, *in planta* evidence of aldaulactone toxicity was compromised due to the experimental barrier when using carrot leaves^39^. In this study, we documented the pathogenicity of *A. dauci* and the aldaulactone toxicity on tobacco leaves. Despite the importance of toxins in the *A. dauci* pathogenicity, the current understanding of its SM production and relevant biosynthetic pathways is still very limited. We provided the first transcriptome of *A. dauci* and the first investigation of SM core gene diversity within *Alternaria* genomes, including *A. dauci*. Finally, we predicted the aldaulactone biosynthesis cluster by a comprehensive phylogenetic analysis and a correlation between aldaulactone production and expression of PKS genes. From the structure of both the toxin biosynthesis cluster and aldaulactone, we proposed a biosynthetic pathway for this toxin (Fig. 9).

The transcriptomic data of *A. dauci* were used, to substantially improve the *A. dauci* genome assembly completeness. *A. dauci’s* genome contained 19 predicted SM clusters, comprising 15 PKS genes, 1 hybrid PKS-NRPS gene, 1 PKS-like gene and 6 NRPS genes. When this bioinformatic prediction in *A. dauci* was compared to the number of PKS gene clusters predicted from 75 Dothideomycetes, *A. dauci* was positioned at the 29th rank^63^. Thus, the number of PKS genes is relatively high, by comparison with a phylogenetically distant *Alternaria* species like *A. brassicicola* (situated at the 62nd rank).

From genomic data available online, we predicted, 55 groups of putative homologous SMs core genes in 21 strains belonging to 19 *Alternaria* species. To our knowledge, no genomic comparative study focused on SMs genes have already been performed at the *Alternaria* genus scale. Moreover, few SM biosynthetic pathways were characterized within *Alternaria*, despite numerous studies using metabolomic profiles and the importance of SMs in the lifestyle of those necrotrophic fungi^39,41,42,62^. Each *Alternaria* genome harbored a different set of SM core genes as shown in Fig. 5, depending on species phylogenic classification. Remarkably, among the 34 PKS-encoding genes, only 7 NR-PKSs were predicted and all were found in the *A. dauci* genome. The dendrograms either based on SM core gene binary repartition or on *Alternaria* spp. phylogenic relationships gave the same three separated clades: strains belonging to the *porri* section (1), or to the *alternata* section (2), and the sole *A. brassicicola* strain (3). *Alternaria* strains inside the *porri* section were also grouped in the same manner by SM core gene criterion and molecular taxonomy (Fig. 5). Contrastingly, *Alternaria* strains in the *alternata* section were not similarly segregated when comparing both analysis tools. Here, patterns of SM core gene were used to help in *Alternaria* taxonomy, as chemotaxonomy analysis. In fact chemotaxonomy was demonstrated to be efficient in *Alternaria* strains/species discrimination in the *porri* section^42,64^. Our SM core gene prediction could be considered as an illustration of a potential secondary metabolome in *Alternaria spp.* independently of culture conditions. In further analyses, the comparison between the presence/absence of SM core genes in *Alternaria* strains and their corresponding metabolic profiles would be useful to identify new biosynthetic pathways.

Among the 55 SM core gene types, 36 had weak similarities to characterized SM genes. This suggests that the relevant metabolites produced may not yet be linked to known biosynthetic pathways or may correspond to unidentified compounds. The remaining 19 SM core gene types presented homologies with genes involved in melanin production and core genes known to be involved in the biosynthesis of pathogenicity factors (Supplementary Table S2)^65^. Twelve and four of these last types are respectively involved in biosynthetic pathways of NHST and HST (AF-, ACR-, AAL- and AM-toxin). Other SM core genes, involved in ferricrocin (intracellular siderophore) and extracellular siderophores, may indirectly play a role in fungal pathogenicity by improving iron uptake^59^.

Interestingly, the SM core genes present in all studied genomes encoded NHST and those present in a single genome encoded HST. Eighteen genes were private to a single *Alternaria* genome, highly suggesting the production of specific SM in the relevant strains. Also, *A. dauci* genome contained genes responsible for the biosynthesis of already described compounds: alternariol, melanin, alternapyrone, aslaniol, tentoxin, ferricrocin, extracellular siderophore, 6-methylsalicylic acid and one of the two genes involved in dehydrocurvularin biosynthetic pathway. Moreover, five of the *A. dauci* genes described here (*AdPKS2*, *AdPKS7*, *AdPKS8*, *AdPKS16* and *AdNRPS6*) were private. Among them, *AdPKS7* and *AdPKS8*, were identified as candidate genes for aldaulactone biosynthesis. Here, transcriptomic data showed expression of *AdPKS1*, *AdPKS3*, *AdPKS6, AdPKS7, AdPKS8*, *AdPKS10, AdPKS13, AdPKS16, AdNRPS1, AdNRPS2* and *AdNRPS3*, highly suggesting the potential ability for *A. dauci* to produce melanin, alternariol, aslaniol, and tentoxin. This would be in line with the fact that aldaulactone was not the unique source of toxicity in *A. dauci* exudates as developed in^39^.

The production of alternariol, alternariol monomethyl ether, zinniol and aldaulactone by *A. dauci* was demonstrated from in vitro cultures^40,42,43^. The tentoxin biosynthesis core gene was present in *A. dauci,* but was not expressed in our culture conditions, nor in previous studies^42,66^. This could be linked to specific culture conditions which did not allow gene expression and SM production, as previously observed for zinniol: in vitro zinniol production is drastically controlled by *A. dauci* culture conditions^40^ and occurs only in long lasting cultures without oxygenation and not in 48 h oxygenated cultures^37^. In order to better understand pathogenicity mechanisms, it would be interesting to decipher the expression patterns of SM core genes during the carrot leaf infection by *A. dauci*. Evidence of other involved pathogenicity factors, like cutinolytic enzyme activity was provided by the observation of subcuticular hyphae^67^. The occurrence of *A. dauci* lesions on carrot and the fungal host range could be partly explained by a joint action of lytic enzymes and a toxin cocktail mixing NHST and HST, as usually observed for necrotrophic fungi.

Concerning *A. dauci* host range, a previous study showed that the fungus was able to produce symptoms and sporulate mainly on different Apiaceae species and secondarily on cultivated species belonging to Brassicaceae, Solanaceae and Valerianaceae^61^. In the present paper, necrotic lesions provoked by *A. dauci* and a toxic effect of aldaulactone, at concentrations found in fungal in vitro cultures were observed on *N. benthamina* leaves. Aldaulactone was thus responsible for necrotic symptoms in a Solanaceae species, which did not belong to the main *A. dauci* host range. This is in contradiction with the idea that aldaulactone could be classified as an HST.

In order to decipher aldaulactone biosynthetic pathway, a retro-biosynthesis approach was used, based on our SM core gene predictions and on the DAL nature of aldaulactone. While several RAL biosynthetic pathways are known among fungi, only one DAL (dehydrocurvularin) biosynthetic pathway was fully described, within two fungal species^47^. Through KS-domain phylogenetic analysis, we highlighted that enzymes responsible for DAL and RAL biosynthesis clustered into two subclades of NR-PKS clade basal to clade I and II and HR-PKS clade I (Fig. 6). Three *A. dauci* candidate genes—*AdPKS7*, *AdPKS8* and *AdPKS14*—clustered within these two subclades. Furthermore, our phylogenetic approach on NR-PKS PT domains showed that *AdPKS14* and *AdPKS8* PT domains clustered with PT domains catalyzing C3–C8 cyclisation.

To assess the candidate genes involved in aldaulactone synthesis, qPCR and HPLC experiments were conducted. Liquid cultures obtained in different conditions led to a wide range of gene expression and aldaulactone production. Different studies have shown an epigenetic regulation of fungal SM gene expression. In particular, drugs affecting epigenetic regulation are used to induce the expression of fungal genes, which are weakly or non-expressed under in vitro conditions^68–71^. In the present study, two histone deacetylase inhibitors, trichostatin A and suberoylanilide hydroxamic acid (SAHA) and a DNA methylation inhibitor (zebularine) were tested, but no significant induction effect on gene expression was observed. These results might be due to the too short exposition time of fungal cultures with the tested drugs^71^.

Among *A. dauci* PKS genes, a significant correlation between the expression level of four genes (*AdPKS-NRPS5*, *AdPKS7*, *AdPKS8* and *AdPKS14*) and aldaulactone production was highlighted. Because of its hybrid nature, *AdPKS-NRPS5* was not further investigated as a candidate for aldaulactone biosynthesis. Here, a very low expression of *AdPKS14,* was observed under growth conditions suitable for aldaulactone production. Furthermore, *AdPKS14* did not cluster with HR-PKS genes and its expression was not correlated with expression of other PKS genes. Contrastingly, *AdPKS7* and *AdPKS8* are good candidates for the biosynthesis of aldaulactone carbon backbone, as both genes belong to the same cluster (cluster 8; Fig. 3). The fact that expression of *AdPKS-NRPS5*, *AdPKS7* and *AdPKS8* is cross correlated, could be interpreted as a co-regulation of their expression associated to the fungal aggressiveness.

According to all results presented in this study, a biosynthetic pathway based on cluster 8 was hypothesized (Fig. 9): AdPKS7 catalyzed the biosynthesis of a reduced aliphatic PK, which was then used by AdPKS8 as a precursor. AdPKS8 extended that PK, and then its PT domain would catalyze a C3–C8 cyclization leading to the benzenediol backbone. At last, the TE domain released the molecule by a ten-membered macrolactone cyclization. Cluster 8 also contained 12 other genes with or without predicted functions. In particular, cluster 8 contained the enzymes required for the addition of a methoxy group on C6: an oxygenase-encoding gene (Fig. 3; rightmost ORF in cluster 8) and a methyl transferase-encoding gene (eleventh ORF from the left). Both enzymes were found to be expressed in transcriptomic data. The oxygenase would add a phenol function to the benzenediol moiety and finally the methyl-transferase gene would methylate this function. The others genes in cluster 8 were also found to be expressed except for the fourth and the sixth ORFs from the left. Those expressed genes include two putative transporter genes, one of them was predicted as a multidrug resistance transporter by fungismash prediction. To our knowledge, aldaulactone was the second example, after dehydrocurvularin^47,72^, of C3–C8 cyclization catalyzed by a PT domain in fungi.

An original integrative approach of retrobiosynthesis combining phylogenetic analysis, gene expression study, and HPLC quantification was used to decipher aldaulactone biosynthetic pathway. This work not only presented the first transcriptomic analysis in *A. dauci,* but also provided the first study of diversity and repartition of SM genes through *Alternaria* genus. Moreover, it revealed the putative biosynthetic pathway of aldaulactone, a DAL type phytotoxin, whose role is crucial in *A. dauci* pathogenicity.

## Methods

### Fungal material

The same four *A. dauci* strains and one *A. brassicicola* strain as in^39^ were used to manage fungal cultures. *A. brassicicola* strain Abra 43 is non-pathogenic on carrot. *A. dauci* strain AUS001 is weakly aggressive, strains FRA001 and FRA017 show intermediate aggressiveness, and strain ITA002 is highly aggressive^61^. Numerous studies were also conducted on strain FRA001^37,39,61,67^. Those strains were collected as described in^61,73^ and freely available from the COMIC collection (COMIC–SFR Quasav, 42 rue Georges Morel, 49070 Beaucouzé cedex, France). A set of 21 genomes belonging to 19 *Alternaria* species were also studied: *A.* *dauci* (BMP 0167-genome reassembly based on FRA001 transcriptome)*, A. alternata* (BMP 0269 and BMP 0238)*, A. arborescens* (BMP 0308), *A. capsici* (BMP 0180), *A. carthami* (BMP 1963), *A. citriarbusti* (BMP 2343), *A. crassa* (BMP 0172), *A. fragariae* (BMP 3062), *A. gaisen* (BMP 2338), *A. limoniasperae* (BMP 2335), *A. longipes* (BMP 0313), *A. mali* (BMP3063 and BMP3064), *A. macrospora* (BMP 1949), *A. porri* (BMP 0178), *A. tangelonis* (BMP 2327), *A. tenuissima* (BMP 0304), *A. tomatophila* (BMP 2032)^55^, *A. brassicicola* (Abra 43)^74^ and *A. solani* (altNL03003)^57^ genomes.

### Fungal growth conditions for transcriptomic samples

FRA001 conidial suspensions for transcriptome were prepared as follows. Three 5 mm mycelial plugs of strain FRA001, previously cultivated on Malt-Agar medium as described in^67^, were placed on a sterile cellophane membrane in a Petri dish (90 mm diameter) containing V8^®^ agar medium [175 mL of vegetable juice V8^®^ (Campbell Soup Company), 3 g of calcium carbonate (CaCO_3_), 15 g of bacteriological agar, final volume adjusted to 1 L with ultrapure water, pH 6.8]. Fungal cultures were incubated in the darkness at 20 ± 2 °C for 10 days. The conidial suspension was prepared by adding 6 mL of 0.1% Tween 20, scraping it with a sterile glass rake, then filtering through two layers of gauze. Conidial density was evaluated on a Malassez cell and adjusted to the required concentration.

Three different culture methods were performed. For the first culture method, a sterile cellophane membrane placed on the surface of a Petri dish (90 mm in diameter) containing “carrot juice” agar medium (200 mL of 100% pure carrot juice, Eckes-Granini, Joker^®^), 3 g calcium carbonate (CaCO_3_), 15 g bacteriological agar, final volume adjusted to 1 L with ultrapure water, pH 6.8) was inoculated with 2 mL of conidial suspension containing 2 × 10^5^ conidia. This culture was then incubated for 24 h at 22 °C in the darkness. For the second and third culture methods, 5 × 10^6^ conidia were inoculated in 100 mL of V8^®^ liquid medium placed in a 250 mL Erlenmeyer flask and incubated for 24 h at 22 °C in the darkness. One culture was maintained with 125 rpm checking, the other one without shaking. Whatever the culture method, the collected germinated conidia were immediately immersed into liquid nitrogen and then stored at − 80 °C until RNA extraction.

### RNA extraction and mRNA library construction

Fungal material was mechanically disrupted by grinding in a mortar in the presence of liquid nitrogen. Total RNA was subsequently extracted using the RNA Nucleospin II^®^ kit (Macherey Nagel, Germany) according to the manufacturer instructions. RNA extracts were then purified by precipitation using 0.1 volume of sodium acetate (3 M, pH 5.2) and 2.5 volumes of cold absolute ethanol (− 20 °C), vigorously mixed and incubated overnight at − 20 °C. Extracts were centrifugated for 1 h at 15,000 g and 4 °C and the supernatant was discarded. The pellet was washed with 500 µL of 75% ethanol. A centrifugation at 15,000 g and 4 °C for 30 min was performed to remove ethanol. The pellet was dried and then dissolved in 30 µL of “RNAse-free” ultrapure water. Evaluation of RNA integrity and concentration was performed by the Experion^®^ automatic electrophoresis system generating a RNA quality indicator (RQI) and concentration data. RNA with a RQI between 7 and 8 were selected.

### Illumina sequencing and de novo assembly

Preparation of RNA-seq library from a pool of fungal RNA, from the tree cultural methods described above, RNAseq protocol and de novo assembly were performed by NGS Services Fasteris (Plan-les-Ouates, Switzerland). cDNA sequencing was conducted on an Illumina HiSeq2000 platform using 100 bp paired-end sequencing strategy. Adapter sequences were removed. The reads were then assembled de novo using the Velvet (1.2.07) software with the additional module Oases^75,76^. A first functional annotation was performed by research of unigenes by clustering the top-hit from BLASTX searches in NR database of NCBI^77^. An InterProScan analysis with Hmmpfam, blastProDom, FPrintScan, ProfileScan applications was performed using Blast2GO^78^.

### Improvement of *A. dauci* genome assembly

The publicly available genome of *A. dauci*^55^ contig datas were used for further scaffolding. AGOUTI was used to stitch genome contigs together based on BWA mediated alignment of transcriptome paired short reads on genome. Ns were added when sequences are unknown^56,79^. A second scaffolding was performed using the CSAR online tool with the “NUCmer on nucleotides” option and *A. solani* genome as reference^57^. A structural annotation was processed by the AUGUSTUS online tool^80^ on the new genome assembly. The gene prediction was done on both strands allowing few alternative transcripts and based on *Botrytis cinerea* as model organism. GenomeQC was used to check assembly contamination and evaluate the metrics and completeness with *Pezizomycotina* BUSCO dataset^81^.

### PCR and sequencing of gaps

To manage gene and cluster reconstruction, PCR amplification of gaps between two sequences was performed. For that purpose, genomic DNA from FRA001 mycelium was extracted according to^82^. Primers used are described in Supplementary Table S4. The amplification reactions with relevant primers were performed in a mix containing 50 mM KCl, 1.5 mM MgCl_2_, 10 mM Tris–HCl (pH 9.0), 0.1% Triton X-100, 0.2 mmol of each dNTP, 0.4 µmol of each primer, 50 ng of genomic DNA and 1 U of Taq polymerase (Promega) in a final volume of 50 µL. The amplification products were purified and sequenced by Eurofins Genomics (Ebersberg, Germany). Sequences were manually reassembled.

### Transcriptome reference based reconstruction

RNA-Seq reads quality was assessed using FasQC^83^. This tool produces statistics, such as average and range of the sequence quality values per base across the reads, GC content, over-represented sequences. Those reads were trimmed using Trimmomatic^84^ with first a cut off the end of read when the quality is under 20 and a drop of reads with average quality lower than 20 (TRAILING:20; AVGQUAL:20). Subsequently, only reads with a minimum length of 20 bp were kept (MINLEN: 20). The paired-end libraries of reads were mapped to *A. dauci* reassembled genome using HISAT2 tool (Galaxy Version 2.1.0 + galaxy3). The reads were further assembled using Stringtie and Cufflinks^58,85^ and the results were merged.

### SM core gene and cluster prediction

Putative *PKS* and *NRPS* gene clusters were predicted from transcriptomic and genomic data using computational tools specialized in gene identification of fungal SM, such as antiSMASH (fungiSMASH version)^86,87^ and SMURF^88^. AntiSMASH (fungiSMASH version) prediction was performed on the 21 *Alternaria* genomes. The PKS and NRPS domains were predicted from the deduced unigene protein using the NCBI Conserved Domain Search and the Hidden Markov Models obtained from PFAM^89^. Presence or absence of each gene was checked in all analyzed genomes by tBlastN research. If the sequences showed more than 80% of similarity in 80% of their length, they were put in the same set of genes. For *A.* *dauci* genome CSAR assembly, SMURF was also used to predict SM clusters with AUGUSTUS structural annotation. When minimal domains were detected, i.e. A-C-PCP for NRPS and KS-AT-ACP for PKS, the relevant genes were renamed using “*AdNRPS”* or “*AdPKS*” as a prefix and a number. This numbering followed the same order than the sequence numbers in the genome.

### Phylogenetic analysis of PKS

A phylogenetic tree was generated with sequences of KS domain predicted using PFAM from a set of Dothideomycetes’ PKS sequences. This set contains the PKS sequences predicted in *A. dauci,* all the Dothideomycetes’ PKS proteins collected from SwissProt protein database (UniProt reviewed database^90^) in^60^ and PKS proteins involved in benzenediol lactone biosynthesis^33,47,53^. Fungal accession numbers, KS domain sequences and known products are listed in Supplementary Table S3. The protein sequences of the KS domain were aligned using MegaX^91^ with ClustalW^92^. The tree was constructed using a maximum parsimony model with 1000 random bootstraps to test for the significance of the resulting topology followed by Tree Bisection-Reconnection (TBR) branch swapping. Bootstrap values under 50 were interpreted as non-significant, bootstrap values between 50 and 90 as weak support and bootstrap values higher than 90 were interpreted as strong support. The tree was rooted using FAS (P49327) of *Homo sapiens* and FASN (P12276) of *Gallus gallus* sequences.

The regio-selectivity of cyclization catalyzed by the PT domain of NR-PKS was determined according to the phylogenetic analysis protocol described in^24^, using at least three representative PT domains for each group^27^. All sequences used for phylogenetic analysis were available in Supplementary Table S3.

An *Alternaria* phylogenetic tree was generated from sequences of four housekeeping genes—*Alternaria* major allergen gene (*Alt a 1*), glyceraldehyde-3-phosphate dehydrogenase (*gapdh*), translation elongation factor 1-alpha (*tef1*) and RNA polymerase second largest subunit (*rpb2*)—found in the set of the 21 *Alternaria* genomes mentioned above. The sequences of each gene were aligned, manually adjusted and then concatenated. The alignment of concatenated sequences was generated with MAFFT v. 7 (http://mafft.cbrc.jp/alignment/server/index.html). Findmodel (http://www.hiv.lanl.gov/content/sequence/findmodel/findmodel.html) was used to choose the nucleotide substitution model. Bayesian analyses were performed with MrBayes v. 3.2.6 (http://www.phylogeny.fr/one_task.cgi?task_type=mrbayes) on the concatenated sequences aligned dataset. A GTR model with gamma-distributed rate variation was used and a Markov Chain Monte Carlo analysis was performed with 10,000 generations from a random tree topology. RAxML v. 0.9.0 was additionally run on the concatenated sequences aligned dataset to performed a maximum-likelihood analysis including 1000 bootstrap replicates.

### Expression patterns of *A. dauci* putative PKS genes by RT-qPCR and HPLC analysis

Liquid cultures of *A. dauci* were obtained by inoculation of 100 mL of medium with 3 mycelial agar plugs in 250 mL Erlenmeyer flasks and growth at 24 °C in the darkness as previously described^39^. Two kinds of experiments were performed. On the one hand, different strains (AUS001, FRA001, FRA017 and ITA002) were grown in stirred Potato Dextrose Broth (PDB, 24 g L^−1^) medium at 125 rpm for 60 h. On the other hand, FRA001 strain was grown under different conditions as described in Table 2. Two different media were used, PDB (24 g L^−1^) and Vogel minimal medium^93^. All cultures were conducted in three replicates separated in time. Briefly, after incubation, culture medium and mycelium were collected by filtration^39^. The mycelium was stored at − 80 °C until RNA extraction. Organic extracts were obtained from filtered culture medium by liquid–liquid extraction with ethyl acetate and dried^37,39^. Absolute quantification of aldaulactone by HPLC was performed on organic extracts from culture filtrates as described in^39^.

**Table 2:** *A. dauci* FRA001 strain liquid culture growth conditions.

Condition name	Culture medium	DMSO final concentration (%)	Additional compound (final concentration)	Culture duration	Shaking (rpm)
Vogel	Vogel^90^	0	None	60 h	125
Zebularine	PDB	0	Zebularine (25 µM)	60 h	125
DMSO	PDB	0.1	None	60 h	125
Trichostatin	PDB	0.1	Trichostatin A (0.5 µM)	60 h	125
SAHA	PDB	0.1	SAHA (250 µM)	60 h	125
PDB	PDB	0	None	60 h	125
21D	PDB	0	None	21 d	None

Total RNA was isolated from mycelium by grinding in a mortar with the lysis buffer according to the manufacturer instructions (NucleoSpin^®^ RNA Plus kit,Macherey–Nagel). RNAs were further treated with the Turbo DNA-free kit (Ambion^®^, ThermoFisher scientific). Quality assessment was performed using a NanoDrop spectrophotometer. When needed, RNA purification was conducted as previously described above. Reverse transcription (RT) was performed on 1 µg of RNA diluted in a final volume of 9 µL RNase-free ultrapure water and heated for 3 min at 80 °C. The RT reaction was performed on heated RNA in a total volume of 30 µL containing 10 pmol oligo dT(15), 0.1 µg of random hexamer, 1 × RT buffer, 0.5 mM dNTP, 200 U of M-MLV Reverse Transcriptase (Promega). The mix was incubated for one hour at 37 °C and finally 10 min at 80 °C. cDNAs were diluted ten-fold with RNase free water and stored at − 20 °C until use.

Primers for PKS genes and housekeeping genes (*tef1*, *Alt a 1*, and *gapdh*) were designed using Perlprimer^94^ based on the predicted gene sequences (Supplementary Table S4). All qPCR reactions were conducted from 2 µL of cDNA obtained as described above in a reaction volume of 10 μL containing 1× master mix (Promega) and 0.1 µM of each primer in RNase-free ultrapure water. Melting curves were checked to assess the specificity of primers. An equimolar pool of cDNAs was realized and four ten-fold dilutions (10 to 10^4^) of this pool were then used as standards. To determined primer efficiency, real-time PCR reactions were performed on standards using StepOnePlus™ Real-Time (RT) qPCR System (Applied Biosystems). When needed, for genes expressed at very low level, cDNA fragments were amplified by PCR using the GoTaq^®^ Flexi DNA Polymerase PCR kit (Promega) in a 25 μL reaction volume (1 µL of diluted cDNA, 1× GoTaq^®^ Flexi Buffer, 1.5 mmol L^-1^ MgCl_2_, 0.2 mM of each dNTP, 0.2 mM of each primer and 0.5 U of GoTaq^®^ DNA Polymerase). PCR reactions were conducted with the following parameters: 2 min at 95 °C, followed by 30 cycles (95 °C, 1 min; 60 °C, 15 s; 72 °C, 30 s) and finally 2 min at 72 °C. The PCR amplifications were then purified using a Nucleospin gel and PCR clean-up kit according to the manufacturer's protocol (Macherey–Nagel). The purified cDNAs were eluted in a final volume of 15 μL of elution buffer (5 mM Tris–HCl, pH 8.0). The cDNAs concentration in ng μL^−1^ was measured using a Nanodrop spectrophotometer, then the copy number of cDNAs per microliter was calculated. A final concentration of 0.5 × 10^5^ cDNA copies µL^−1^ was added to cDNA pool before dilutions in ten-fold series. qPCR experiments were performed on 384 wells microplate with each experimental condition tested in triplicates. For each primer mix, a negative control (water), a positive control (genomic DNA of *A. dauci*) and serial cDNA dilutions as standards were used in the same plate than all the analyzed samples. The deposition of cDNA and reaction mixture (primer and master mix) in the microplates was carried out using a Zephyr Compact Liquid Handling Workstation (CaliperLife Science) robot controlled by the Caliper Life Maestro Workstation Software. The qPCR plates were monitored by Biorad CFX384 machine. For each *AdPKS* gene in each sample, the Ct value was compared with a mean of the Ct values obtained from the three reference genes. A method based on^95^ was used to calculate expression ratios. All data were compared using a Kruskal–Wallis test.

### Assessment of *A. dauci* pathogenicity and aldaulactone toxicity on tobacco leaves

Model plant *N. benthamiana* seeds were obtained in compliance with relevant institutional, national, and international guidelines and legislation. In the greenhouse, *N.* *benthamiana* seedlings were transplanted 15 days after sowing in 75 centiliters pots containing potting soil (Substrate 5, Klasmann^®^) and axillary branches were removed. Plants were maintained with a 16-h photoperiod, a day/night temperature of 23/19 °C and a relative humidity of about 70%. The three first true developed leaves of six-week-old plants were used for two experimental conditions consisting of leaf inoculation or leaf infiltration. First, a conidial suspension of FRA001 strain was prepared according to^61^ with a final concentration of 1000 conidia mL^−1^. For plant infection, the conidial suspension was spread with a sterile brush on the abaxial leaf side. A mock inoculation was also performed. Second, the organic phase obtained from filtrates of ITA002 liquid culture (100 mL) or PDB medium (100 mL, negative control) was used for leaf infiltration. The filtrates were obtained as described in^39^ and dissolved in DMSO to obtain a maximal concentration of 0.1% in a final volume of 100 mL (volume adjusted with ultrapure water). As a reference, a 0.1% DMSO solution (negative control) and two aldaulactone solutions at final concentrations of 12.5 mg L^−1^ and 50 mg L^−1^ were prepared. The different filtrates or solutions were infiltrated at five points on a same leaf by pressing a 1 mL needleless syringe on the abaxial leaf side. Two biological replicates were performed. Nine days after infiltration, the leaves were collected, scanned at a resolution of 300 dpi using an Epson Perfection 3200 Pro flatbed image scanner. Images obtained were analyzed using the FIJI software^96^ to quantify necrosis area. For inoculated plants with FRA001, isolation from symptomatic leaf pieces was realized on PDA medium (amended with streptomycin 500 mg L^−1^) and incubated as described in^61^. DNA was extracted from mycelial colonies and from FRA001 following the protocol described in^82^. PCR experiments were conducted on three targeted sequences: *ITS, tef1* and *IGS* (primer sequences in Supplementary Table S4). The amplification products were purified and sequenced by Eurofins Genomics. Sequences were manually reassembled and then aligned with relevant sequences obtained from FRA001.

## Supplementary Information

Supplementary Data S1.

Supplementary ﻿Data S2.

Supplementary ﻿Data S3.

Supplementary ﻿Data S4.

Supplementary Information.

## References

[1] Stergiopoulos I, Collemare J, Mehrabi R, Wit PJ (2013). Phytotoxic secondary metabolites and peptides produced by plant pathogenic Dothideomycete fungi. FEMS Microbiol. Rev..

[2] Pusztahelyi T, Holb IJ, Pócsi I (2015). Secondary metabolites in fungus-plant interactions. Front. Plant Sci..

[3] Horbach R, Navarro-Quesada AR, Knogge W, Deising HB (2011). When and how to kill a plant cell: Infection strategies of plant pathogenic fungi. J. Plant Physiol..

[4] Walton JD (1996). Host-selective toxins: Agents of compatibility. Plant Cell.

[5] Petrov V, Qureshi MK, Hille J, Gechev T (2018). Occurrence, biochemistry and biological effects of host-selective plant mycotoxins. Food Chem. Toxicol..

[6] Möbius N, Hertweck C (2009). Fungal phytotoxins as mediators of virulence. Curr. Opin. Plant Biol..

[7] Wolpert TJ, Dunkle LD, Ciuffetti LM (2002). Host-selective toxins and avirulence determinants: What’s in a Name?. Annu. Rev. Phytopathol..

[8] Meena M, Samal S (2019). Alternaria host-specific (HSTs) toxins: An overview of chemical characterization, target sites, regulation and their toxic effects. Toxicol. Rep..

[9] Turgeon BG, Baker SE (2007). Genetic and genomic dissection of the Cochliobolus heterostrophus Tox1 locus controlling biosynthesis of the polyketide virulence factor T-toxin. Adv. Genet..

[10] Walton JD (2006). HC-toxin. Phytochemistry.

[11] Wolpert TJ, Macko V (1989). Specific binding of victorin to a 100-kDa protein from oats. Proc. Natl. Acad. Sci..

[12] Keller NP, Turner G, Bennett JW (2005). Fungal secondary metabolism—From biochemistry to genomics. Nat. Rev. Microbiol..

[13] Brakhage AA (2013). Regulation of fungal secondary metabolism. Nat. Rev. Microbiol..

[14] Keller NP (2018). Fungal secondary metabolism: Regulation, function and drug discovery. Nat. Rev. Microbiol..

[15] Shen B (2003). Polyketide biosynthesis beyond the type I, II and III polyketide synthase paradigms. Curr. Opin. Chem. Biol..

[16] Staunton J, Weissman KJ (2001). Polyketide biosynthesis: A millennium review. Nat. Prod. Rep..

[17] Shimizu Y, Ogata H, Goto S (2017). Type III polyketide synthases: Functional classification and phylogenomics. ChemBioChem.

[18] Hashimoto M, Nonaka T, Fujii I (2014). Fungal type III polyketide synthases. Nat. Prod. Rep..

[19] Robbins T, Liu Y-C, Cane DE, Khosla C (2016). Structure and mechanism of assembly line polyketide synthases. Curr. Opin. Struct. Biol..

[20] Herbst DA, Townsend CA, Maier T (2018). The architectures of iterative type I PKS and FAS. Nat. Prod. Rep..

[21] Hertweck C (2009). The biosynthetic logic of polyketide diversity. Angew. Chem. Int. Ed..

[22] Ahuja M (2012). Illuminating the diversity of aromatic polyketide synthases in *Aspergillus nidulans*. J. Am. Chem. Soc..

[23] Chooi Y-H, Cacho R, Tang Y (2010). Identification of the viridicatumtoxin and griseofulvin gene clusters from *Penicillium aethiopicum*. Chem. Biol..

[24] Li Y, Xu W, Tang Y (2010). Classification, prediction, and verification of the regioselectivity of fungal polyketide synthase product template domains. J. Biol. Chem..

[25] Crawford JM (2008). Deconstruction of iterative multidomain polyketide synthase function. Science.

[26] Chooi Y-H, Tang Y (2012). Navigating the fungal polyketide chemical space: From genes to molecules. J. Org. Chem..

[27] Liu L (2015). Bioinformatical analysis of the sequences, structures and functions of fungal polyketide synthase product template domains. Sci. Rep..

[28] Liu L, Zhang Z, Shao C-L, Wang C-Y (2017). Analysis of the sequences, structures, and functions of product-releasing enzyme domains in fungal polyketide synthases. Front. Microbiol..

[29] Du L, Lou L (2010). PKS and NRPS release mechanisms. Nat. Prod. Rep..

[30] Reimer JM, Haque AS, Tarry MJ, Schmeing TM (2018). Piecing together nonribosomal peptide synthesis. Curr. Opin. Struct. Biol..

[31] McErlean M, Overbay J, Van Lanen S (2019). Refining and expanding nonribosomal peptide synthetase function and mechanism. J. Ind. Microbiol. Biotechnol..

[32] Bushley KE, Turgeon BG (2010). Phylogenomics reveals subfamilies of fungal nonribosomal peptide synthetases and their evolutionary relationships. BMC Evol. Biol..

[33] Yun C-S, Motoyama T, Osada H (2015). Biosynthesis of the mycotoxin tenuazonic acid by a fungal NRPS–PKS hybrid enzyme. Nat. Commun..

[34] Farrar JJ, Pryor BM, Davis RM (2004). Alternaria diseases of carrot. Plant Dis..

[35] Ben-Noon E, Shtienberg D, Shlevin E, Vintal H, Dinoor A (2001). Optimization of chemical suppression of *Alternaria dauci*, the causal agent of Alternaria leaf blight in carrots. Plant Dis..

[36] Davis, R. M. Carrot diseases and their management. in *Diseases of Fruits and Vegetables Volume I* 397–439 (Springer, 2004).

[37] Lecomte M (2014). Partial resistance of carrot to *Alternaria dauci* correlates with in vitro cultured carrot cell resistance to fungal exudates. PLoS ONE.

[38] Meena M (2017). Alternaria toxins: Potential virulence factors and genes related to pathogenesis. Front. Microbiol..

[39] Courtial J (2018). Aldaulactone—An original phytotoxic secondary metabolite involved in the aggressiveness of *Alternaria dauci* on carrot. Front. Plant Sci..

[40] Barash I, Mor H, Netzer D, Kashman Y (1981). Production of zinniol by *Alternaria dauci* and its phytotoxic effect on carrot. Physiol. Plant Pathol..

[41] Leyte-Lugo M, Richomme P, Poupard P, Peña-Rodriguez LM (2020). Identification and quantification of a phytotoxic metabolite from *Alternaria dauci*. Molecules.

[42] Pinto, V. E. F. & Patriarca, A. Alternaria Species and Their Associated Mycotoxins. in *Mycotoxigenic Fungi: Methods and Protocols* (eds. Moretti, A. & Susca, A.) 13–32 (Springer, New York, 2017). 10.1007/978-1-4939-6707-0_2. 10.1007/978-1-4939-6707-0_2 27924529

[43] Freeman GG (1966). Isolation of alternariol and alternariol monomethyl ether from Alternaria dauci (kühn) groves and skolko. Phytochemistry.

[44] Wenderoth M (2019). Alternariol as virulence and colonization factor of Alternaria alternata during plant infection. Mol. Microbiol..

[45] Qui JA, Castro-Concha LA, García-Sosa K, Miranda-Ham ML, Peña-Rodríguez LM (2010). Is zinniol a true phytotoxin? Evaluation of its activity at the cellular level against *Tagetes erecta*. J. Gen. Plant Pathol..

[46] Zheng L, Lv R, Huang J, Jiang D, Hsiang T (2010). Isolation, purification, and biological activity of a phytotoxin produced by *Stemphylium solani*. Plant Dis..

[47] Cochrane RVK (2015). Comparison of 10,11-dehydrocurvularin polyketide synthases from *Alternaria cinerariae* and *Aspergillus terreus* highlights key structural motifs. Chembiochem. Eur. J. Chem. Biol..

[48] Kim Y-T (2005). Two different polyketide synthase genes are required for synthesis of zearalenone in *Gibberella zeae*. Mol. Microbiol..

[49] Xie X, Meehan MJ, Xu W, Dorrestein PC, Tang Y (2009). Acyltransferase mediated polyketide release from a fungal megasynthase. J. Am. Chem. Soc..

[50] Zhou H (2010). Enzymatic synthesis of resorcylic acid lactones by cooperation of fungal iterative polyketide synthases involved in hypothemycin biosynthesis. J. Am. Chem. Soc..

[51] Reeves CD, Hu Z, Reid R, Kealey JT (2008). Genes for the biosynthesis of the fungal polyketides hypothemycin from *Hypomyces subiculosus* and radicicol from *Pochonia chlamydosporia*. Appl. Environ. Microbiol..

[52] Wang S (2008). Functional characterization of the biosynthesis of radicicol, an Hsp90 inhibitor resorcylic acid lactone from *Chaetomium chiversii*. Chem. Biol..

[53] Gaffoor I, Trail F (2006). Characterization of two polyketide synthase genes involved in zearalenone biosynthesis in *Gibberella zeae*. Appl. Environ. Microbiol..

[54] Horsman ME, Hari TPA, Boddy CN (2016). Polyketide synthase and non-ribosomal peptide synthetase thioesterase selectivity: Logic gate or a victim of fate?. Nat. Prod. Rep..

[55] Dang HX, Pryor B, Peever T, Lawrence CB (2015). The Alternaria genomes database: A comprehensive resource for a fungal genus comprised of saprophytes, plant pathogens, and allergenic species. BMC Genomics.

[56] Zhang SV, Zhuo L, Hahn MW (2016). AGOUTI: improving genome assembly and annotation using transcriptome data. GigaScience.

[57] Wolters PJ (2018). Gapless genome assembly of the potato and tomato early blight pathogen *Alternaria solani*. Mol. Plant. Microbe Interact..

[58] Chen K-T, Lu CL (2018). CSAR-web: A web server of contig scaffolding using algebraic rearrangements. Nucleic Acids Res..

[59] Oide S (2006). NPS6, encoding a nonribosomal peptide synthetase involved in siderophore-mediated iron metabolism, is a conserved virulence determinant of plant pathogenic ascomycetes. Plant Cell.

[60] Kroken S, Glass NL, Taylor JW, Yoder OC, Turgeon BG (2003). Phylogenomic analysis of type I polyketide synthase genes in pathogenic and saprobic ascomycetes. Proc. Natl. Acad. Sci..

[61] Boedo C (2012). Evaluating aggressiveness and host range of Alternaria dauci in a controlled environment: Evaluating aggressiveness and host range of Alternaria dauci. Plant Pathol..

[62] Lou J, Fu L, Peng Y, Zhou L (2013). Metabolites from Alternaria fungi and their bioactivities. Molecules.

[63] Noar RD, Daub ME (2016). Bioinformatics prediction of polyketide synthase gene clusters from *Mycosphaerella fijiensis*. PLoS ONE.

[64] Andersen B, Dongo A, Pryor BM (2008). Secondary metabolite profiling of *Alternaria dauci*, *A. porri*, *A. solani*, and *A. tomatophila*. Mycol. Res..

[65] Kimura N, Tsuge T (1993). Gene cluster involved in melanin biosynthesis of the filamentous fungus *Alternaria alternata*. J. Bacteriol..

[66] Li Y-H (2016). Putative nonribosomal peptide synthetase and cytochrome P450 genes responsible for tentoxin biosynthesis in *Alternaria alternata* ZJ33. Toxins.

[67] Boedo C (2008). Impact of carrot resistance on development of the Alternaria leaf blight pathogen (*Alternaria dauci*). Eur. J. Plant Pathol..

[68] Kjærbølling I, Mortensen UH, Vesth T, Andersen MR (2019). Strategies to establish the link between biosynthetic gene clusters and secondary metabolites. Fungal Genet. Biol..

[69] Macheleidt J (2016). Regulation and role of fungal secondary metabolites. Annu. Rev. Genet..

[70] Pfannenstiel BT, Keller NP (2019). On top of biosynthetic gene clusters: How epigenetic machinery influences secondary metabolism in fungi. Biotechnol. Adv..

[71] Li C-Y (2020). Natural products development under epigenetic modulation in fungi. Phytochem. Rev..

[72] Xu Y (2013). Characterization of the biosynthetic genes for 10, 11-dehydrocurvularin, a heat shock response-modulating anticancer fungal polyketide from *Aspergillus terreus*. Appl. Environ. Microbiol..

[73] Iacomi-Vasilescu B (2004). In vitro fungicide sensitivity of Alternaria species pathogenic to crucifers and identification of *Alternaria brassicicola* field isolates highly resistant to both dicarboximides and phenylpyrroles. Crop Prot..

[74] Belmas E (2018). Genome sequence of the necrotrophic plant pathogen *Alternaria brassicicola* Abra43. Genome Announc..

[75] Schulz MH, Zerbino DR, Vingron M, Birney E (2012). Oases: Robust de novo RNA-seq assembly across the dynamic range of expression levels. Bioinform. Oxf. Engl..

[76] Zerbino, D. & Birney, E. Velvet: Algorithms for De Novo short read assembly using De Bruijn Graphs. *Genome Res.* gr.074492.107 (2008) 10.1101/gr.074492.107. 10.1101/gr.074492.107 PMC2336801 18349386

[77] blastx: search protein databases using a translated nucleotide query. https://blast.ncbi.nlm.nih.gov/Blast.cgi?PROGRAM=blastx&PAGE_TYPE=BlastSearch&LINK_LOC=blasthome.

[78] Quevillon E (2005). InterProScan: Protein domains identifier. Nucleic Acids Res..

[79] Li H, Durbin R (2009). Fast and accurate short read alignment with Burrows-Wheeler transform. Bioinform. Oxf. Engl..

[80] Stanke M, Morgenstern B (2005). AUGUSTUS: A web server for gene prediction in eukaryotes that allows user-defined constraints. Nucleic Acids Res..

[81] Manchanda N (2020). GenomeQC: A quality assessment tool for genome assemblies and gene structure annotations. BMC Genomics.

[82] Goodwin, D. C. & Lee, S. B. Microwave miniprep of total genomic DNA from fungi, plants, protists and animals for PCR. *BioTechniques* **15**, 438, 441–2, 444 (1993). 8217156

[83] Andrews, S. FastQC: A quality control tool for high throughput sequence data. https://www.bioinformatics.babraham.ac.uk/projects/fastqc/ (2010).

[84] Bolger AM, Lohse M, Usadel B (2014). Trimmomatic: A flexible trimmer for Illumina sequence data. Bioinformatics.

[85] Chen K-T (2018). CSAR: A contig scaffolding tool using algebraic rearrangements. Bioinformatics.

[86] Blin K (2019). antiSMASH 5.0: Updates to the secondary metabolite genome mining pipeline. Nucleic Acids Res..

[87] Medema MH (2011). antiSMASH: Rapid identification, annotation and analysis of secondary metabolite biosynthesis gene clusters in bacterial and fungal genome sequences. Nucleic Acids Res..

[88] Khaldi N (2010). SMURF: Genomic mapping of fungal secondary metabolite clusters. Fungal Genet. Biol. FG B.

[89] El-Gebali S (2019). The Pfam protein families database in 2019. Nucleic Acids Res..

[90] Apweiler R (2004). UniProt: The universal protein knowledgebase. Nucleic Acids Res..

[91] Kumar S, Stecher G, Li M, Knyaz C, Tamura K (2018). MEGA X: Molecular evolutionary genetics analysis across computing platforms. Mol. Biol. Evol..

[92] Thompson, J. D., Gibson, T. J. & Higgins, D. G. Multiple sequence alignment using ClustalW and ClustalX. *Curr. Protoc. Bioinforma.* **00**, 2.3.1–2.3.22 (2003). 10.1002/0471250953.bi0203s00 18792934

[93] Vogel HJ (1956). A convenient growth medium for Neurosporacrassa. Microbial Genet. Bull..

[94] Marshall OJ (2004). PerlPrimer: Cross-platform, graphical primer design for standard, bisulphite and real-time PCR. Bioinform. Oxf. Engl..

[95] Pfaffl MW (2001). A new mathematical model for relative quantification in real-time RT–PCR. Nucleic Acids Res..

[96] Rueden CT (2017). Image J2: ImageJ for the next generation of scientific image data. BMC Bioinform.

