# Supplementary material for: Characterization of NRPS and PKS genes involved in the biosynthesis of SMs in Alternaria dauci including the phytotoxic polyketide aldaulactone
Source: Sci Rep. 2022 May 17;12:8155. doi: 10.1038/s41598-022-11896-0 (PMC9114375; doi:10.1038/s41598-022-11896-0)
Supplement: Supplementary file 5 — Supplementary Information. [file 41598_2022_11896_MOESM5_ESM.docx]

# Supplementary material

Supplementary Data S1: Blast hits for putative *Alternaria dauci* SM biosynthetic cluster genes. BLASTp analysis was done for each gene of each cluster using the non-redundant protein sequence database on NCBI. The results are presented by cluster, relative gene position in the cluster are indicated with respect to the core gene position. Top ten hits are indicated with species where they are found, description of the gene, maximum bitscore, percent query cover, E-value, percent sequence identity, and Gene bank accession number.

Supplementary Data S2: SM core genes predicted in twenty *Alternaria* genome from ^51,54,71^ using antiSMASH, SM core gene presence/absence matrix and top Hit BLAST synthesis for each SM core gene type.

Supplementary Data S3: KS and PT domain sequences for phylogenic analysis from both known and uncharacterized NR-PKSs.

Supplementary Data S4: List of primers used in the present study to amplify *A. dauci* gDNA or cDNA
